# DNA Methylation Is Responsive to the Environment and Regulates the Expression of Biosynthetic Gene Clusters, Metabolite Production, and Virulence in *Fusarium graminearum*

**DOI:** 10.3389/ffunb.2020.614633

**Published:** 2021-01-15

**Authors:** Christopher Bonner, Amanda Sproule, Owen Rowland, David Overy, Rajagopal Subramaniam

**Affiliations:** ^1^Department of Biology, Carleton University, Ottawa, ON, Canada; ^2^Ottawa Research and Development Centre, Agriculture and Agri-Food Canada, Ottawa, ON, Canada

**Keywords:** *Fusarium graminearum*, bisulfite sequencing, DNA methyltransferase, Metaboilte profiling, RNA-Seq

## Abstract

Histone modifications play a significant role in the regulation of biosynthetic gene clusters (BGCs) in the phytopathogen *Fusarium graminearum*, by contrast, epigenetic regulation by DNA methyltransferases (DNMTs) is less documented. In this study, we characterized two DNMTs (FgDIM-2 and FgRID) in *F. graminearum*, with homologies to “Deficient in methylation” (DIM-2) and “Repeat-induced point (RIP) deficient” (RID) from Neurospora. The loss of DNMTs resulted in not only a decrease in average methylation density in the nutrient-poor, compared to nutrient-rich conditions, but also differences in the genes expressed between the WT and the DNMT mutant strains, implicating the external environment as an important trigger in altering DNA methylation patterns. Consequently, we observed significant changes in the regulation of multiple BGCs and alterations in the pathogenicity of the fungus.

## Introduction

In eukaryotes, epigenetics can be described broadly as changes in geneactivity, without changes to the underlying genetic code (Cavalli and Heard, 2019). This definition encompasses a wide variety of mechanisms including histone modification and DNA methylation (Wang et al., 2017; Cavalli and Heard, 2019). Both of these mechanisms alter the structure of DNA and serve as a bridge between the environment and the genotype, giving rise to changes in phenotype through regulation of gene expression. Epigenetic mechanisms have been implicated in a wide variety of processes such as X-chromosome inactivation, initiation and development of cancers, and cellular, and development processes in mammals and plants (Gendrel and Heard, 2014; Friedrich et al., 2019). Studies have also revealed the importance of epigenetics in gene regulation in fungi, both at the individual gene and at the genome level through chromatin remodeling with the formation of heterochromatin structures (Strauss and Reyes-Dominguez, 2011). This process is largely dictated by post-translation modifications of histones by methylation, and acetylation, and the methylation of DNA. Detailed studies in mammals have revealed that both degree and specificity of methylation of lysine residues on histone proteins impact “chromatin states” to determine gene activities (Black et al., 2012). More importantly, both regulation and dysregulation of enzymes such as histone methyltransferases and demethylases involved in post-translational modifications of histone residues contribute to the etiology of disease states (Kalish et al., 2014).

In contrast to mammalian studies, epigenetic studies are at a nascent state in phytopathogenic fungi. However, the consequences of changes in both histone and DNA methylation marks have been shown to impact the lifecycle of fungi. A study by Connolly et al. showed that almost a third of the genome of the cereal pathogen *Fusarium graminearum* possessed methylation marks on lysine 27 of the histone protein 3 (H3K27me3) (Connolly et al., 2013). An absence of these methylation marks relieved the genome to express an additional 14% of total genes that were previously “silent” and many of these genes were associated with BGCs. Changes in DNA methylation have also been recognized to play a role in both genome defense in *N. crassa* and *Ascobolus immerses* and in fungal development in the rice pathogen *Magnaportha oryzae* (Honda et al., 2012; Jeon et al., 2015). More recently, So et al. showed that colony sectoring *in vitro*, observed in the chestnut blight fungus *Cryphonectria parasitica*, is a result of epigenetic modifications through DNA methyltransferases (DNMTs) (So et al., 2018). Other studies have implicated DNA methylation in fungal virulence and stress tolerance (Wang et al., 2017).

DNA methylation involves the deposition of a methyl group from the donor *S-*adenosyl-methionine to the fifth position on a cytosine base (5mC) by a DNA methyltransferase enzyme. DNMTs in fungi were first described in *N. crassa* where the DNMT designated “Deficient in methylation” (DIM-2) is responsible for 5mC methylation and the second DNMT “Repeat-induced point (RIP) deficient” (RID) is essential for genome defense (Kouzminova and Selker, 2001; Freitag et al., 2002; Aramayo and Selker, 2013). Phylogenetic analyses suggested that both DIM-2 and RID belong to the same monophyletic DNMT1 family and that RID evolved before DIM-2 (Bewick et al., 2019). The extent of 5mC methylation in fungal species is wide-ranging from an imperceptible (<0.1%) in *Schizosaccharomyces pombe*, and *Aspergillus flavus*, to intermediate (0.22% in *M. oryzae*, 0.48% in *Cordyceps militaris*, 1.5% *Neurospora crassa*, 3.9% *Cryphonectria parasitica*), to high (>40% in *Tuber melanosporum*) (Antequera et al., 1984; Ponger and Li, 2005; Liu et al., 2012; Montanini et al., 2014; Jeon et al., 2015; Xin et al., 2019).

The mycotoxigenic fungus *Fusarium graminearum* is the causal agent of Fusarium head blight disease (FHB) of wheat. Due to the economic impact on world agriculture trade, the fungus has attained notoriety and is the subject of many studies. Despite considerable attention, no studies to date have investigated the role of DNMTs on fungal development and pathogenicity in *F. graminearum*. This study clarifies the role of DNMTs in this phytopathogen and provides insight into their role in the regulation of genes involved in secondary metabolism and pathogenicity.

## Materials and Methods

### Strains and Culture Conditions

*F. graminearum* strain NRRL29169 (DAOM233423) was obtained from the Canadian Collection of Fungal Cultures (DAOMC). Macro-conidia was used as the starting inoculum for all experiments and was produced in liquid CMC (carboxy-methyl-cellulose) according to Schreiber et al. (2011). Mycelia were grown in either Preferred Nutrient medium (PN) (56 mM NH_4_Cl, 8.1 mM MgSO_4_ 7H_2_O, 0.23 mM FeSO_4_·7H_2_O, 14.7 mM KH_2_PO_4_, 2 g L^−1^ Peptone, 2 g L^−1^ Yeast extract, 2 g L^−1^ malt extract and 111 mM glucose) or in non-preferred nutrient (NPN) medium (6.2 mM Putrescine di-hydrochloride, 22 mM KH_2_PO_4_, 0.8 mM MgSO_4_·7H_2_O, 85.6 mM NaCl, 116.8 mM sucrose, 108.6 mM glycerol, pH 4.0).

### Identification of DNA Methyltransferases in *F. graminearum*

Two DNA methyltransferases in *F. graminearum* were identified through reciprocal BLASTp to *Neurospora tetrasperma DIM-2* (Deficient in methylation) and *RID* (Repeat Induce Defective) as well as through genome annotation database searches. No additional DNA methyltransferases were identified by either method. Methyltransferase domains were examined using InterproScan (Zdobnov and Apweiler, 2001). Protein sequences for 35 eukaryotic DNA methyltransferases were identified from Yang et al. (2016) and obtained from NCBI (https://www.ncbi.nlm.nih.gov/protein/) for comparative analysis under accession numbers: DMT (*E. coli* O26:H11), 608747058; DmtA (*A. oryzae*), 317148994; DmtA (*A. niger*), 145250405; DmtA (*A. nidulant*), 28208637; DmtA (*A. kawachii*), 358374015; DmtA (*A. fumigatus*), 846909269; DmtA (*N. fischeri*), 119467548; DmtA (*A. clavatus*), 119398323; DmtA (*A. flavus*), 220695028; Dim-2 (*M. robertsii*), 629717833; Dim-2 (*B. bassiana*), 667652773; Dim-2 (*N. tetrasperma*), 350287792; Masc1 (*A. immersus*), 2558956; RID (*N. crassa*), 20531189; RID (*N. tetrasperma)*, 20531193; Dnmt1 (*A. thaliana*), 15239810; Dnmt1 (*D. rerio*), 190338613; Dnmt1 (*H. sapiens*), 195927037; Dnmt1 (*R. norvegicus*), 214010196; Dnmt1 (*S. scrofa*), 73853882; Dnmt1 (*M. musculus*), 148693193; Dnmt1 (*O. aries*), 57164173; Dnmt1 (*X. laevis*), 148225023; Dnmt1 (*B. mori*), 112983430; Dnmt2 (*A. thaliana*), 18420929; Dnmt2 (*S. frugiperda*), 406868804; Dnmt2 (*S. scrofa*), 242253856; Dnmt2 (*D. elanogaster*), 116007318; Dnmt3 (*D. rerio*), 190337984; Dnmt3 (*A. mellifera*), 298677086; Dnmt3 (*D. pulex*), 321467881; Dnmt3B (*D. rerio*), 70887603; Dnmt3A (*D. rerio*), 688595232; CMT (*A. thaliana*), 15222449; CMT (*M. domestica*), 658309668 (Supplementary Figure 1B). Phylogenetic relationships were inferred using the Neighbor-Joining method to generate an appropriate starter tree (Saitou and Nei, 1987), followed by the Maximum-Likelihood method under the WAG model for protein substitution (Whelan and Goldman, 2001) and the default settings of CLC-Genomics Workbench (Qiagen Bioinformatics v9.5.3) to create the final tree (Supplementary Figure 1A). Each clade was supported by bootstrap consensus inferred from 1,000 replicates (Felsenstein, 1985).

### Construction and Characterization of *FgDim-2* and *FgRid* Mutant Strains

Single mutant strains of DNA methyltransferase genes *FgDIM-2* (Δ*FgDim-2*) and *FgRID* (Δ*FgRid*) and a double mutant strain (Δ*FgDim-2*/Δ*FgRid*), were generated by the USER (Uracil-Specific Excision Reagent) friendly cloning system in conjunction with *Agrobacterium tumefaciens* (LBA4404) (Frandsen et al., 2008). A schematic of this protocol can be seen in Supplementary Figure 2. Complementation strains of both single deletion mutants were also created. Select transformants were grown from a single spore, confirmed by PCR and RT-qPCR, and submitted to the Canadian Collection of Fungal Cultures (Supplementary Figure 3). NovaSeq6000 PE100 sequencing was performed on the double deletion mutant to confirm its genetic identity (Supplementary Figure 3C).

### Phenotypic Characterization of *DNMT* Mutant Strains

The mutant strains were characterized for vegetative growth, sexual development, and pathogenicity. Vegetative growth was assessed by dry weight after 3-day growth in a two-step media system. Spores were inoculated at 5,000 sp/mL in PN conditions for 24 h before being transferred to NPN conditions for 48 h. This was repeated twice with six technical replicates for each biological replicate. Differences in mycelial dry weight were assessed using Student's *T*-test. Sexual reproduction was assessed visually by the production of perithecia on carrot agar (Wang et al., 2014) with 8–12 technical replicates per strain and two independent biological experiments. Toxin production was assessed for all strains with three independent experiments and between 18 and 47 technical replicates, following growth in a two-stage media system (Walkowiak et al., 2015). A pathology test was performed for all the strains by point inoculation on a susceptible variety of wheat (*cv*. Roblin) (Walkowiak et al., 2015). Three independent experiments were performed with 18–62 wheat spikelets.

### Whole Genome Bisulfite Sequencing (WGBS) and Analyses

Whole-genome bisulfite sequencing was performed according to pre-established methods at the Genome Quebec sequencing facility using the Illumina HiSeq X PE150 system on genomic DNA isolated from both WT *F. graminearum* and the double deletion mutant strain, Δ*FgDim-2*/Δ*FgRid*. Genomic DNA was isolated from both 24 h preferred nutrient (PN) and 6 h non-preferred (NPN) conditions. Raw sequencing reads were checked for quality and trimmed before alignment in CLC-Workbench (v12.0) using the “Map bisulfite reads to reference” tool. DNA methylation was assessed using the “Call methylation levels” tool. Sites identified as methylated were then subjected to a binomial test in Excel to correct for both errors in sequencing and incomplete bisulfite conversion: = BINOM.DIST (Methylated Coverage, Context Coverage, Error Rate, False). Methylated sites that passed the binomial correction (*P*-value ≤ 0.05) were selected for further analysis. The degree of methylation was described in terms of level and density, which were defined as the number of 5mC reads divided by the total number of reads covering each site and by the number of 5mC sites per 10 kb, respectively (So et al., 2018). The sequence reads from the WGBS can be accessed through NCBI accession PRJNA 587083 (reviewer link: https://dataview.ncbi.nlm.nih.gov/object/PRJNA587083?reviewer=rst5c59jhj5hvvbutslfq67083).

WGBS results were validated using Sanger sequencing as described in So et al. (2018). In brief, mycelia from the WT and the double mutant strains were grown for 6 h under NPN conditions in triplicate. The genomic DNA was extracted using the EZNA Fungal DNA kit (Omega bio-tek Cat#D3390-02) and bisulfite-converted using the EpiMark Bisulfite Conversion kit (New England Biolabs E3318S). The converted DNA was PCR amplified using primers (Loci_Contig1_9033981-9034488 F/R and Loci_Contig6_127178-127396 F/R) specific for converted DNA and for sites which were predicted by WGBS to have methylation in all samples. Amplified products were cloned into the Zero Blunt™ Topo™ vector (ThermoFisher Scientific Cat#450245) and three to five clones corresponding to each locus were sequenced using Sanger sequencing using the M13F/R primers. The repetitive elements comprised all repeats identified, including interspersed repeats, transposable elements, and tandem repeats/satellites. Repeats were identified using RepeatMasker and RepeatModeler (http://www.repeatmasker.org).

### RNA-Sequencing and Gene Expression Analyses

Total RNA was extracted using TRIzol (Invitrogen) from mycelia grown in triplicates under two different environmental conditions: 24 h in PN and NPN conditions. For each condition, 5,000 spores/mL of each WT and Δ*FgDim-2*/Δ*FgRid* were used. The RNA obtained from TRIzol extractions was purified using the Stratec RNA cleanup kit according to the manufacturer's instructions. The RNA samples were assessed for quality and quantity on the Agilent 2100 Bioanalyser and RNA samples with an Integrity Number of >6.5 were sent to Genome Quebec for sequencing on either the Illumina HiSeq 2500 PE125 (PN) or the Illumina HiSeq 4000 PE100 (24 h NPN). Sequencing reads were assessed for quality and trimmed in CLC-Genomics Workbench (v12.0). Gene expression analysis using the “RNAseq Analysis” function was performed on sequencing reads mapped to gene coding sequences. Differentially expressed genes (DEGs) were considered those with a *P*-value of ≤ 0.05 and a fold change of ≤ −2 or ≥ 2. All sequence mapping was performed against *F. graminearum* NRRL29169 CDS (GenBank Accession: SPRZ00000000). RNA sequencing data were deposited with GEO accession GSE140030 (https://www.ncbi.nlm.nih.gov/geo/query/acc.cgi?acc=GSE140030).

For the real-time quantitative PCR analysis (RT-qPCR), total fungal RNA was extracted from mycelia the WT and Δ*FgDim-2*/Δ*FgRid* strains grown in NPN from 24 h as described before. The two genes, FGSG_02322 and FGSG_09595 were chosen to validate RNA sequencing data due to their high transcript abundance and role as part of two BGCs. The RNA was reverse transcribed and the RT-qPCR reactions were performed in triplicate using Power SYBR Green PCR Master Mix (Applied Biosystems, Thermofisher, Canada) and the QuantStudio3 qPCR machine (Applied Biosystems, Thermofisher, Canada) according to the manufacturer's instructions. The expression of each gene was quantified using primers sets FGSG_02322 qPCR F/R and FGSG_09595 qPCR F/R and normalized against two housekeeping genes using primer sets FGSG_09530 (β-tubulin) qPCR F/R and FGSG_16627 (GAPDH) qPCR F/R (Supplementary Table 5).

### Metabolic Profiling

Quadruplicate cultures of WT and Δ*FgDim-2*/Δ*FgRid* were grown in flasks at 5,000 spores/mL with shaking at 170 rpm in 5% PN:95% NPN conditions for 14 days and metabolomics analysis was performed (Shostak et al., 2020). Mycelium was harvested, freeze-dried, and extracted using 25 mL ethyl acetate (EtOAc) with shaking at 200 rpm and ambient temperature for 2 h. Mycelium was removed by centrifugation and the solvent was dried *in vacuo*. Culture extracts were reconstituted in 1:1 acetonitrile: water to a concentration of 500 μg/mL and a 5 μL aliquot was analyzed using a Thermo Scientific Dionex Ultimate 3000 ultra-high-performance liquid chromatography system coupled to a Thermo LTQ Orbitrap XL high-resolution mass spectrometer (Thermo Scientific, Waltham, MA, USA). Chromatography was performed using a Kinetex C18 column (100 Å, 2.1 × 50 mm, 1.7 μm; Phenomenex, Torrance, CA, USA) maintained at a temperature of 30°C. The mobile phase consisted of water containing 0.1% formic acid (A) and acetonitrile containing 0.1% formic acid (B) and the flow rate was 0.350 mL^−1^. The gradient started at 5% B, held for 0.5 min, and increased to 95% B over 4.5 min, and maintained at 95% B for 3.5 min. The gradient was returned to starting conditions and re-equilibrated for 5 min. The HRMS was operated in ESI^+^ mode scanning a *m/z* range of 100–2,000 Da at a resolution of 30,000 using the following parameters: sheath gas 40, auxiliary gas 5, sweep gas 2, spray voltage 4.0 kV, capillary temperature 320Â°C, capillary voltage 35 V and tube lens 100 V (MS2 scans were acquired using CID at 35 eV).

Data preprocessing was performed using MZmine2 v2.52 [Cell Unit, Okinawa Institute of Science and Technology (OIST), Onna, Okinawa, Japan] with a mass detection threshold of 1 × 10^5^ and using the ADAP algorithm (with a minimum group size of 5 and a group intensity threshold/minimum highest intensity of 1E5). Chromatographic deconvolution was achieved using the local minimum search method with the following parameters: chromatographic threshold 80%, search minimum in RT range 0.5 min, minimum relative height 35%, minimum absolute height 1E5, a minimum ratio of peak/top edge 0.5, and peak duration range 0.0–10.0 min. Isotopes were removed and the data were aligned using the Join aligner with a 5.0 ppm *m/z* tolerance, a 0.2 min RT tolerance, and a *m/z* vs. RT weight of 5:1. All variables in the aligned feature list were gap-filled using the peak finder method and normalized to the total ion current. Multivariate and univariate statistical analyses were performed on the resulting data matrix in RStudio using the “muma” package with autoscaling and figures were generated using the “ggplot2” package. A list of significant variables (*p* < 0.05) with log_2_ fold changes > 1 and < −1 was generated. Variables were grouped to reduce the dimensions of the data set if their associated retention times were within ± 0.02 min and their Pearson correlation coefficients were ≥ 0.85. Variables (mass features) were annotated by matching with an in-house *Fusarium* metabolite database and any matches within 5 ppm were annotated and associated psuedomolecular ions (i.e., protonated mass, adducts, and neutral losses) confirmed by consulting the RAW data. MassWorks^TM^ software (v5.0.0, Cerno Bioscience) was used to improve spectral accuracy and confirm the molecular formulas of annotated ions. The sCLIPS searches were performed in dynamic analysis mode with elements C, H, N, and O allowances set at minimum 1 and maximum 100. The charge was specified as 1, mass tolerance was set to 5 ppm and the profile mass range was −1.00 to 3.50 Da.

## Results

### DNA Methyltransferases Alter Fungal Development and Pathogenicity

Two genes encoding putative DNMTs have been identified in *F. graminearum*, however, no functional studies were conducted (Bewick et al., 2019). The first DNMT (FGSG_10766) from *F. graminearum* shared 48.3% protein identity with the *Neurospora tetrasperma* protein *Nt*DIM-2 and the second DNMT protein from *F. graminearum*, FGSG_08648 shared 35.6% identity with the *Nt*RID protein (Supplementary Table 1). Maximum-likelihood phylogenetic analysis of 35 DNMTs from 29 eukaryotic species (16 fungi, 12 mammals, 1 plant) and 1 prokaryotic species (*E. coli*) was undertaken to understand the evolutionary origins of DNMTs identified in *F. graminearum* (Supplementary Figures 1A,B). Gene expression profiling from various environmental conditions had shown that both *FgDNMTs* are constitutively expressed at low levels (Subramaniam et al., 2015). Five *F. graminearum* strains: deletion of FGSG_10766 (Δ*FgDIM-2*), deletion of FGSG_08648 (Δ*FgRID*), a double mutant strain (Δ*FgDim-2*/Δ*FgRid*), and two complementation strains (Δ*FgDim-2/FgDIM-2* and Δ*FgRid*/ *FgRID*) were constructed to assess their role in fungal development, the ability to produce the mycotoxin 15-acetyldeoxynivalenol (15-ADON) and their virulence in wheat (Supplementary Figures 2, 3).

**Figure 1:**
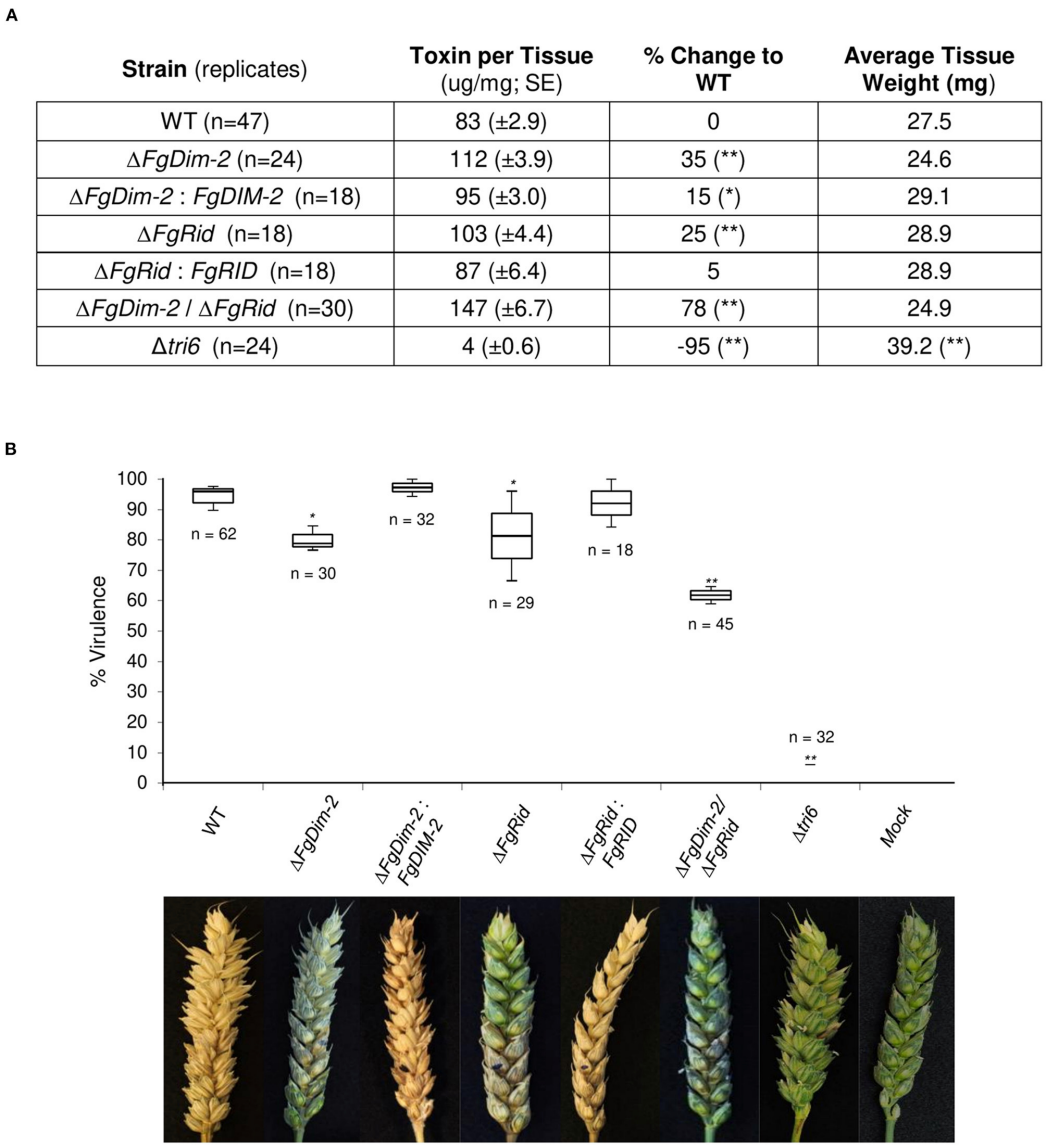
The DNMTs in *F. graminearum* regulate 15-ADON production and virulence. **(A)** Toxin production was quantified by HPLC in three independent experiments with standard error. Strains significantly differing in toxin production compared to WT are denoted by a single asterisk (*0.05 > *p*-value > 0.01), or a double asterisk (**p-value < 0.01) as determined by Student's two-tailed *t-*test. **(B)** Both methyltransferase mutants reduce infection on wheat. The box plot represents the interquartile range with a median virulence of a minimum from three independent experiments with a total of “n” inoculated heads. Strains significantly different in virulence compared to WT are denoted by a single asterisk (*0.05 > *p*-value > 0.01), or a double asterisk (**p-value < 0.01) as determined by Student's two-tailed *t-*test. Images are taken 15 days post-infection; the infection site is denoted by a black dot. Mock heads were inoculated with water. The Δ*tri6* strain was used as a non-pathogenic control.

Fungal development was assessed by growth in axenic culture by dry weight and production of perithecia (sexual structures) on carrot agar (Supplementary Figure 4). While there were no differences in the growth among the strains, the mutant strains on average produced fewer perithecia compared to the wild-type (WT) strain (Supplementary Figure 4). We assessed the ability of the mutant strains to produce the mycotoxin 15-ADON in culture. Both the single mutant strains produced more toxin (25–35%) compared to the wild-type (WT) strain, which was mostly complemented by the respective WT genes (Δ*FgDim-2/FgDIM2* and Δ*FgRid/FgRID*; Figure 1A). Moreover, we also observed an additive effect in the double mutant strain Δ*FgDim-2*/Δ*FgRid* with a 78% increase in 15-ADON production compared to the WT strain (Figure 1A). The mutant strains were also assessed for virulence by pathology tests performed with a susceptible wheat variety “Roblin” (Figure 1B). The single mutants displayed a decrease in virulence by ~15% compared to the WT strain, however; we also observed an additive effect with the double mutant strain Δ*FgDim-2*/ Δ*FgRid* with a ~30% reduction in virulence compared to WT (Figure 1B). These results indicate that *Fg*DNMTs contribute to the mycotoxin production, and virulence and suggest that increased capacity to produce 15-ADON by the mutant strains does not necessarily have an impact on the virulence of the fungus. Thus, for pathogenicity, factors other than DON are influenced by the action of DNMTs.

### Methylation Density Is Dynamic Between Environmental Conditions

With the demonstration of phenotypes associated with the DNMTs, we were interested to know the role of DNMTs on DNA methylation in *F. graminearum*. We performed whole-genome bisulfite sequencing (WGBS), which enabled analysis of individual methylation sites as well as genome-wide methylation patterns in both the wild-type *F. graminearum* and the double mutant Δ*FgDim-2*/Δ*FgRid* strains. DNA methylation was assessed in axenic cultures grown in preferred nutrient (PN) for 24 h and compared to cultures grown in non-preferred nutrient (NPN) conditions for 6 h. Non-preferred conditions are conducive for the production of an array of secondary metabolites and therefore any changes in the methylation patterns can be related to the activation of secondary metabolite gene clusters (Gardiner et al., 2009). The bisulfite-converted samples were sequenced by Illumina HiSeqX and the reads were mapped to the reference genome (GenBank Accession: SPRZ00000000). Mapping efficiencies ranged from 87 to 91% resulting in 41–63 million mapped reads (Supplementary Table 2). This represented a median coverage of 196× with regions of zero coverage amounting to <0.0094% of the reference (~1 kb).

We assessed methylation level (proportion of methylation at a single site in a population) in three cytosine contexts (CpG; CHG; CHH) to be on average between 4 and 5%, with only a small variation between strains and environmental conditions (Supplementary Table 3). To validate the observations from the WGBS results, two 5mC sites in Contig 1 (position numbers: 9034013, and 9034142) three 5mC sites in Contig 6 (position numbers 127218, 127244, and 127351) conserved in both strains and environmental conditions were selected for analysis. The regions of interest were PCR amplified from the sodium bisulfite-converted genomic DNA, cloned, and subjected to Sanger sequencing. All the cytosine sites identified as methylated by WGBS were shown to be protected from bisulfite conversion to uracil/thymine, thus validating the WGBS experiments. Regarding methylation density (#5mC/Total #s of genomic C), only minor differences were observed at the genome-wide level (Average range: 1.9–2.4%) in both of the strains and between the two environmental conditions (Supplementary Table 4). However, in contrast to the methylation level, methylation density displayed a significant preference for the asymmetrical CHH cytosine compared to either CpG (*p*-value 1.2 E-8) or CHG (*p-*value 2.7 E-9) contexts across both genotypes and environmental conditions (Supplementary Table 4).

**Figure 2:**
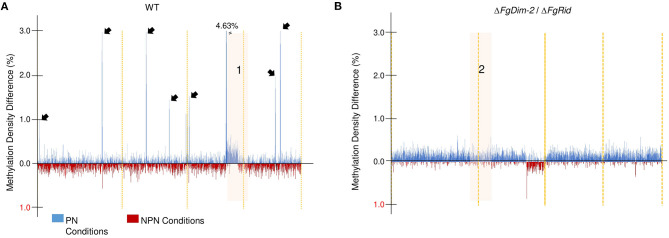
Genome-wide DNA methylation density differences of the WT and the Δ*FgDim2*/Δ*FgRid F. graminearum* strains in the two environmental conditions. DNA methylation was binned in 10 kb regions and the average methylation density for each bin was plotted on the *X*-axis across the genome. Bins with greater average methylation density in PN conditions are in blue; bins with greater average methylation density in NPN conditions are in red. **(A)** Average binned DNA methylation in the WT strain was more prominent in PN conditions with regions of dense DNA methylation punctuating the genome (denoted by black arrows). The region “1” display more/dense methylation in PN condition compared to NPN; a higher resolution image of this region is shown in Supplementary Figure 6. **(B)** Average binned DNA methylation in the Δ*FgDim2*/Δ*FgRid* strain. A region of dense methylation difference between PN and NPN conditions is denoted as “2” and a higher resolution image of this region is shown in Supplementary Figure 6.

To further investigate methylation changes in the two environmental conditions, we constructed a differential methylation density map between the PN and NPN conditions in both the WT and the mutant strain Δ*FgDim-2*/Δ*FgRid* (Figure 2). The methylation sites in the whole genome were binned in 10 kb sections, and the average methylation for each bin was calculated. The differences in methylation in each bin between the PN and NPN conditions are shown (Figure 2). In the WT strain, methylation differences between the two conditions were punctuated with regions of high DNA methylation density in the PN condition (black arrows, Region 1; Figure 2A). Except for region 1, which spanned 1.67 Mbp at the end of chromosome III, the areas of increased methylation density marked by arrows were predominantly gene-poor and had low GC content (Figure 2A). On the other hand, the differential methylation pattern in the double mutant strain Δ*FgDim-2*/Δ*FgRid* was distinct from the WT strain (Figure 2B). Except for region 2 encompassing 2.38 Mbp at the end of chromosome II, we observed overall increased methylation in the PN condition compared to the NPN condition. However, at the genome-wide level, the increase was not statistically significant (Region 2, Figure 2B). Higher resolution images of regions 1 and 2 highlights the dynamic changes in methylation density that occurs in specific regions that are affected by environmental conditions (Supplementary Figure 6). Regions 1 and 2 contained 646 and 923 genes, respectively and although a large number of these genes in both regions were associated with metabolism; none of them were linked to any BGC (Supplementary Data Sheet 1). These results suggested that activities of DNMTs have a significant impact on methylation patterns within a short period (i.e., 6 h) and potentially influence the phenotypic outcomes.

**Figure 3:**
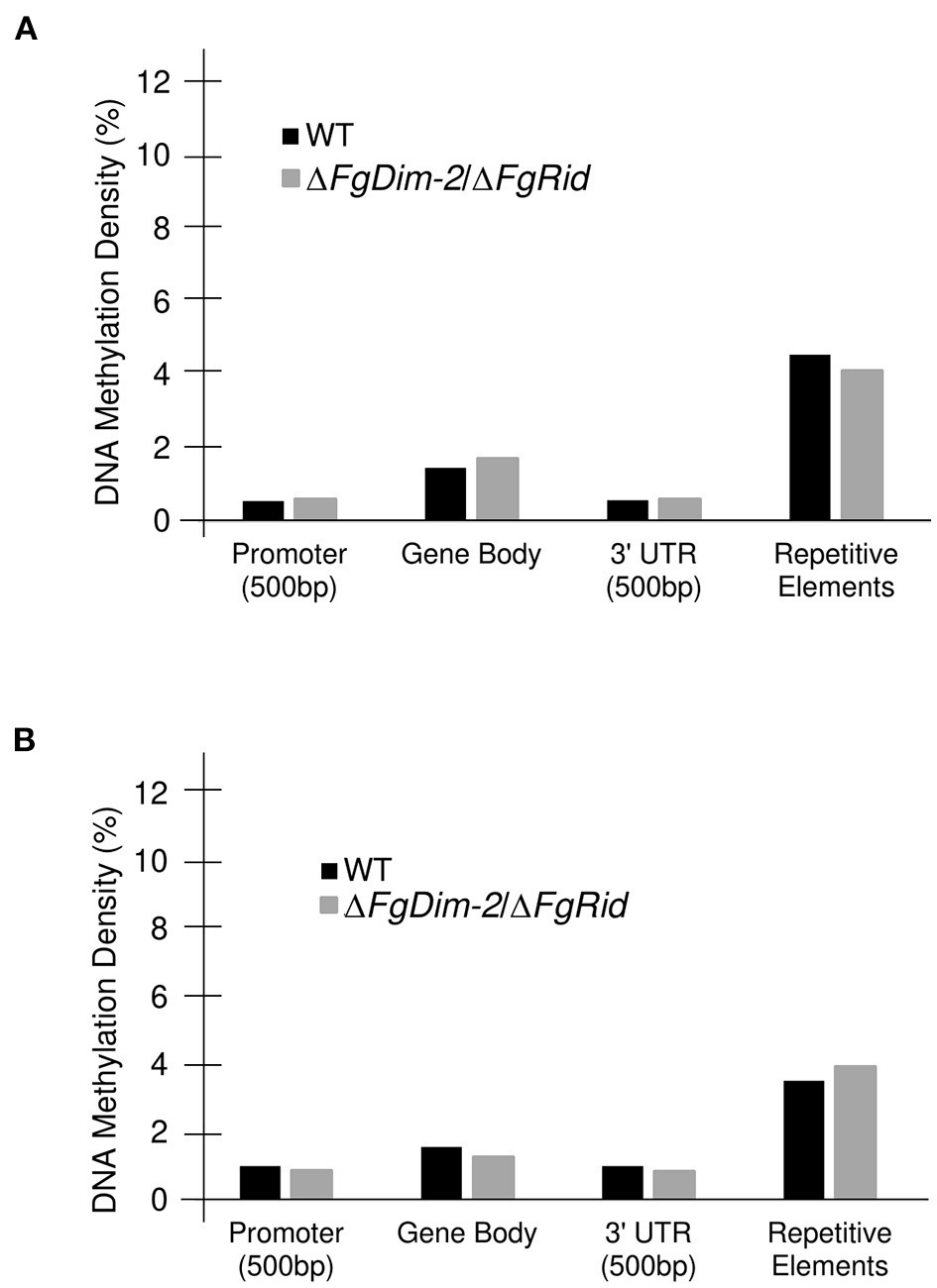
Methylation density differences in the genomic features between the WT and Δ*FgDim-2*/Δ*FgRid F. graminearum* strains. DNA methylation density (# methylated cytosine/total cytosine) for each genomic feature; a 500 bp promoter region, the gene body, a 500 bp 3′UTR for each gene as well as for repetitive elements was calculated for all 14,190 genes. **(A)** DNA methylation density in WT and Δ*FgDim-2*/Δ*FgRid* strains under PN conditions for 24 h. **(B)** Under NPN conditions for 24 h.

We also assessed methylation density among various genomic features. In comparison to the entire genome, methylation density was below average (Average range: 1.9–2.4%) in both the promoter and the 3′UTR regions, on par in the gene body, and above average in the repetitive elements in both environmental conditions (Figure 3). A drop in the methylation density of repetitive elements was observed in the WT strain when the culture was switched from the PN to the NPN conditions (Compare Figures 3A,B). Statistical analysis (Student's *t*-test; two-tailed distribution) revealed no significant differences between the two genotypes within the same nutrient condition. However, a comparison of average % methylation in each repetitive element between the nutrient conditions in the WT strain showed a significant difference (WT-NPN to WT-PN; *p*-value: 0.01). No significant differences were observed in the mutant strain between the nutrient conditions (DKO-NPN to DKO-PN; *p*-value: 0.6). We identified a total of 50 repeat elements with the majority (32) classified as interspersed repeats of unknown origin (Supplementary Data Sheet 2). Of the remainder, three were classified as rRNA pseudogenes, and five as transposable elements, and among the transposable elements identified, two were classified as an LTR GypsyERV, one as a Tc1-Mariner-like PogoGroup Fot1 and two were Line dependent retrotransposons SINEs. (Supplementary Data Sheet 2). No major differences were observed in methylation density between the Watson and Crick DNA strands, however, regions near chromosome boundaries, and with low GC/gene count exhibited an above-average density of methylation sites (Supplementary Figure 5). Overall, our analysis revealed that DNA methylation in the *F. graminearum* genome is regulated by DNMTs. Furthermore, external environments such as nutrition availability can also potentially influence DNA methylation.

### Environmental-Induced Gene Expression Is Modified by DNMTs

Since we observed a dynamic shift in methylation patterns orchestrated by DNMTs between the two nutrient conditions, we were interested to know if these changes impacted gene expression in the fungus in those two environments. Therefore, RNA-sequencing was performed with mycelia from both the WT and the double mutant strain Δ*FgDim-2*/Δ*FgRid* grown in PN and NPN conditions for 24 h.

**Figure 4:**
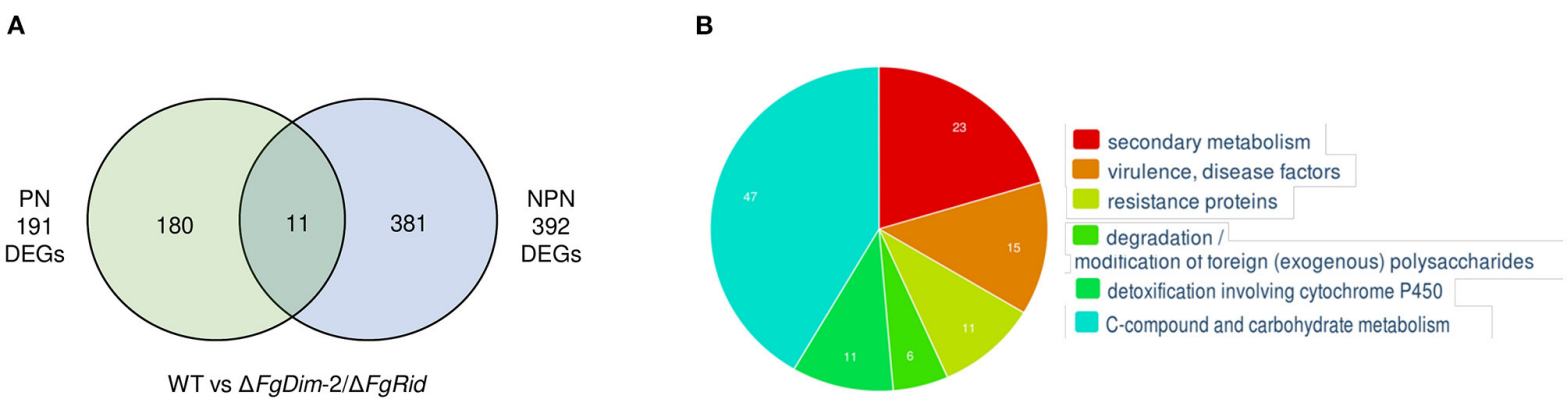
Differentially expressed genes (DEGs) between the WT and the Δ*FgDim-2*/Δ*FgRid F. graminearum* strains grown in PN and NPN conditions for 24 h. **(A)** A Venn diagram representing the number of DEGs expressed two-fold or greater between the two strains under PN and NPN conditions (*p-*value ≤ 0.05). A total of 191 genes were identified to be differentially expressed under PN conditions between the WT and Δ*FgDim-2*/Δ*FgRid* strains, of which 180 were unique to the PN condition. Under NPN conditions, 392 DEGs were identified between the WT and Δ*FgDim-2*/Δ*FgRid* strains, 381 of which were unique to the NPN conditions. A total of 11 DEGs overlapped between the two environmental conditions. Details are presented in Supplementary Data Sheet 2. **(B)** Functional categorization of DEGs under NPN conditions between the two strains as defined by FunCat (Priebe et al., 2015). Six categories were identified as functionally enriched—the proportion of query (DEGs) genes is significantly greater (FDR *p-*value ≤ 0.05) than would be expected by the proportion of all genes in the genome fitting into this functional category. A description of these enriched genes can be found in Supplementary Data Sheet 2.

Analyses between the WT and mutant strains, grown in the PN condition revealed 191 differentially expressed genes (DEGs) (Figure 4). These 191 DEGs were classified using FungiFun, KEGG (Kyoto encyclopedia of genes and genomes) pathway, and by comparisons to known secondary metabolite gene clusters in *F. graminearum* (Adpressa et al., 2019). No enriched categories were identified by FungiFun, however, KEGG (Kyoto encyclopedia of genes and genomes) analysis revealed 30 pathways linked with the DEGs and among them, 12 and 7 DEGs were associated with primary and secondary metabolism (SM), respectively (Supplementary Data Sheet 3). Of the 191 DEGs, 17 genes were associated with 14 known secondary metabolite gene clusters in *F. graminearum* with the majority (11 clusters) downregulated in the mutant strain such as clusters associated with pigment production [C28: carotenoids (2 DEGs) and C53: perithecial pigments (1 DEG)] (Supplementary Data Sheet 3) (Adpressa et al., 2019; Shostak et al., 2020). The DEGs that were upregulated in the mutant strain was linked to clusters C12 (2 DEGs) and C02 (1 DEG), with cluster C02 encoding NPS8, the NPS linked to the production of gramillins (Bahadoor et al., 2018; Adpressa et al., 2019).

In contrast to PN, a greater number of DEGs (392) were observed in cultures grown in the NPN condition, in agreement with the differential methylation density observed between these two environmental conditions (Figure 4). The functional categorization of 392 DEGs by FungiFun showed six significantly enriched categories, with secondary metabolic genes among the most prevalent (Supplementary Data Sheet 3). The six categories were comprised of secondary metabolism (1.2; 23 genes), virulence/disease factors (32.05.05; 15 genes), resistance proteins (32.05.01; 11 genes), degradation of foreign (exogenous) polysaccharides (32.10.07; 6 genes), detoxification involving cytochrome P450 (32.07.01; 11 genes) and C-compound and carbohydrate metabolism (1.05; 47 genes). The KEGG analysis identified 53 metabolic pathways that were present in the biosynthesis of several secondary metabolites (fgr01110; 18 genes). Comparison to known *F. graminearum* SM gene clusters identified 41 DEGs associated with 20 distinct BGCs (Supplementary Data Sheet 3). The genes that were downregulated in the mutant strain were identified as part of clusters C09 (2 DEGs), C13 (8 DEGs; aurofusarin), C27 (1 DEG), C35 (1 DEG), C40 (3 DEGs), C44 (1 DEG), C49 (5 DEGs; butenolide), C52 (4 DEGs), and C72 (1 DEG). Clusters with upregulated genes in the mutant strain included C07 (3 DEGs), C10 (1 DEG), C12 (1 DEG), C18 (1 DEG; orcinol), C24 (1 DEG; fusariumdiene), C31 (2 DEGs), C38 (1 DEG), C51 (1 DEG), C67 (1 DEG), and C69 (1 DEG). Although the majority of these BGCs were identified by only one or two DEGs under NPN conditions, we also observed co-expression of several genes associated with a BGC: eight genes of the aurofusarin gene cluster (C13) and five genes of the butenolide gene cluster (C49) (Supplementary Data Sheet 3).

A qRT-PCR analysis of select genes FGSG_02322 and FGSG_09595 validated the RNA-seq data. FGSG_02322 encodes a transporter, aurT in the aurofusarin BGC and FGSG_09595 encodes an efflux pump (Supplementary Figure 6). Although, regions 1 and 2 showed dramatic methylation density differences and contained no BGCs, only 13/647 of genes in region 1 and 37/923 of genes in region 2 genes were differentially expressed (Supplementary Data Sheet 1). Together, the data suggested that regulation of SM in *F. graminearum* is highly dependent on the surrounding environmental conditions, and the regulation is likely due to modifications of chromatin structure promoted by the DNMTs.

### Secondary Metabolite Production Is Affected by DNMTs

**Figure 5:**
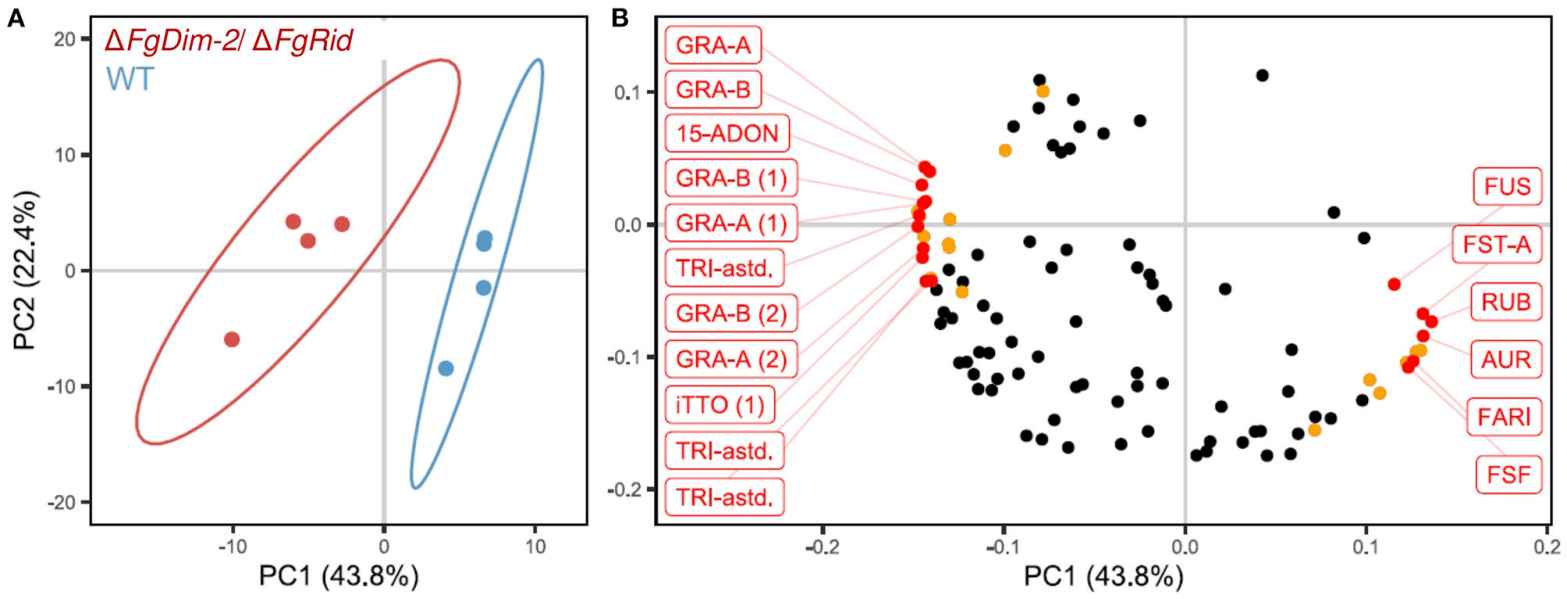
Principal component analysis of metabolites in the extracts of the wild-type (WT) and the mutant Δ*FgDim-2*/Δ*FgRid* strains. **(A)** PC1/PC2 score plot denotes distinct sample clustering along PC 1; ellipses represent 95% confidence intervals. **(B)** PC1/PC2 loadings plot of mass features responsible for sample clustering; colored dots represents metabolite features with significant fold differences (*p*-value < 0.05) observed in univariate analysis (Supplementary Data Sheet 4). Annotated mass features were highlighted in red (15-ADON, 15-acetyl-deoxynivalenol; AUR, aurofusarin; FARI, fusarium asexual reproduction inducer; FST-A, fusaristatin A; FUS, fusarin-associated; GRA, gramillin; RUB, rubrofusarin; TRI-astd, richothecene-associated; iTTO, isotrichotriol; FSF, fuscofusarin; metabolite structural analogs are designated by parentheses).

To further investigate the role of DNMTs upon the regulation of secondary metabolism in *F. graminearum*, both WT and Δ*FgDim2*/Δ*FgRid* mutant strains were cultured in 5%PN:95%NPN conditions (14 days), and mycelial extracts were profiled using UPLC-HRMS for metabolomics analysis (Shostak et al., 2020). The UPLC-HRMS profiles were deconvolved into a collection of mass features (representing eluent retention time and observed mass/charge ratio; RT_*m/z*). To remove the potential bias of differential ionization rates between various metabolites during multivariate data modeling, the data matrix of observed mass features were autoscaled before Principal Component Analysis (PCA). The resulting PCA data model showed that mycelial extracts of WT and Δ*FgDim2*/Δ*FgRid* strains formed two distinct clusters (represented by 95% confidence ellipses explaining 44% of the variance in mass feature expression (PC1) (Figure 5A). All subsequent PC's in the PCA modeled variance associated with differences in mass feature signal intensity between individual strain replicates. The analysis also revealed that mass features with negative loadings values were associated with the Δ*FgDim2*/Δ*FgRid* strain cluster, while mass features with positive loadings values were associated with the WT strain cluster (Figure 5B). The PCA loading values were combined with results from univariate pairwise comparisons of non-scaled mass feature relative intensity values to generate a list of mass features with significant fold changes (*p*-value > 0.05) between WT and Δ*FgDim2*/Δ*FgRid* clusters (Figure 5B; Supplementary Data Sheet 4).

A total of 12 mass features (having positive loading in PC 1) were significantly enriched in the WT strain, including pigmented molecules such as aurofusarin, rubrofusarin, and fuscofusarin along with fusarins, fusaristatin A, and the metabolite FARI (Fusarium asexual reproduction inducer (Qi et al., 2018), also co-named as tricinolone (Adpressa et al., 2019). The differential pigment production was consistent with observed culture phenotypes (i.e., loss of pigment production in Δ*FgDim2*/Δ*FgRid* strain compared to WT) as well as the RNA-Seq and RT-qPCR experiments that showed downregulation of a majority of the genes in the aurofusarin gene cluster in the double mutant strain (Supplementary Data Sheet 3; Supplementary Figure 7). Butenolide was not detected in the UPLC-HRMS profiles as it is highly polar and eluted with the injection volume in the chromatography conditions used for profiling, which was diverted to waste as it contained a considerable amount of medium components (i.e., sugars, salts, etc.) that would have compromised the HRMS source.

In culture extracts of the Δ*FgDim2*/Δ*FgRid* strain, the relative signal intensity of a total of 23 mass features in UPLC-HRMS profiles was found to be significantly enriched compared to the WT strain with mass features associated with 15-ADON (and related biosynthetic intermediates), fusaoctaxins, and gramillins (Supplementary Data Sheet 4). This result also corroborated observations from our axenic culture experiments, where an overall increase of 15-ADON accumulation was observed in the mutant strain (Figure 1A). The UPLC-HRMS^n^ experiments were carried out for all significant mass features, where similarities in MS^2^ and MS^3^ pseudomolecular ion fragmentation patterns led to the identification of several fusaoctaxin and gramillin analogs associated with Δ*FgDim2*/Δ*FgRid* mutants (Shostak et al., 2020). The data collected from HRMS^n^ experiments allowed us to assign putative molecular structures for the two new gramillin molecules; herein, assigned the names, gramillin E, and F (Supplementary Figure 8). Both gramillin E and F are cyclic lipopetides, structurally similar to gramillin A and B, respectively that differ with the presence of a dehydroalanine residue incorporated in place of serine in the cyclic peptide portion of the molecule (Bahadoor et al., 2018). Two additional gramillin analogs were also observed that shared identical molecular weight and MS^n^ fragmentation patterns to that of gramillin A and B but eluted slightly later in the chromatographic run. We hypothesize that in these two analogs, leucine is likely substituted with isoleucine in the cyclic peptide moiety of the molecule; however, proof of this substitution will require purification and confirmation by NMR experiments, which was outside of the scope of this manuscript. It is important to note that the gramillin BGC (C02) was found to be upregulated in expression in the Δ*FgDim2*/Δ*FgRid* strain compared to the WT strain in PN conditions.

Overall, we observed a strong correlation between the BGC expression and metabolite accumulation from mycelium extracts of WT and Δ*FgDim2*/Δ*FgRid* strains. *F. graminearum* has the potential to produce a considerable number of secondary metabolites; only 21 of its 76 hypothesized BGC have known, structurally determined metabolite products (Adpressa et al., 2019). Metabolite annotation was possible for 60% of the discriminatory mass features observed from metabolomics analyses; where the remaining mass features likely represent metabolites not previously associated with *F. graminearum*.

## Discussion

Fungal species are highly adaptable in their interactions with the natural environment. The fungal lifestyle, in particular pathogenic fungi, demands a high degree of plasticity to adapt to constant pressure from the hostile host organisms (Fernandes and Carter, 2020). Epigenetic modifications offer a mechanism where quick changes in the environment could be translated into adaptive phenotypes. This study implicated the two DNA methyltransferases *Fg*DIM-2 and *Fg*RID in *F*. *graminearum* in fungal development, pathogenicity, and in the regulation of secondary metabolism. Genetic loss mutants of *DNMT*s displayed altered phenotypes such as changes in secondary metabolite production (pigmentation and virulence factors) and reduced virulence on susceptible wheat (Roblin). A similar *Dim-2*/*Rid* double mutation decreased pathogenicity by 66% in the entomopathogenic fungus *Metarhizium robertsii* (Wang et al., 2017).

The WGBS profiles confirmed that both DNMTs contribute to DNA methylation and the DNA methylation pattern is dynamic in response to environmental conditions (Figure 2). The loss of *DNMT*s resulted in a region-specific decrease in average methylation density in the NPN condition but also differences in the genes expressed between WT and Δ*FgDim2*/Δ*FgRid* strains, implicating the external environment as an important trigger in altering DNA methylation patterns. Thus, the disruption of an organism's ability to methylate DNA has a direct impact on its ability to transition between different metabolic states.

DNA methylation in fungi exerts a suppressive effect after the initiation of gene transcription, making heterochromatin refractory to transcription; where, in the absence of DNA methylation, transcriptional activity may be capable of overcoming and preventing the formation of heterochromatin (Rountree and Selker, 2010). In *N. crassa*, the adapter protein HP1 acts as a fulcrum in heterochromatin formation and maintenance by recruiting several protein complexes to trimethylated-histone marks (H3K9me3) that are associated with different, yet redundant mechanisms: DNA methylation by the HP1/DIM-2 complex, histone deacetylation by HCHC complex (HP1/CDP-2/HAD-1/CHAP), and histone demethylation to protect heterochromatin boundaries by the putative DMM complex (HP1/DMM-1/DMM-2) (Honda and Selker, 2008; Rountree and Selker, 2010; Honda et al., 2012). Reciprocal to the differential secondary metabolite expression observed in our study between WT and Δ*FgDim2*/Δ*FgRid* mutant strains, deletion of the *HP1* ortholog in *F. graminearum* (Δ*Hep1*) resulted in a 3-4 fold increase in aurofusarin production (and higher transcript levels of aurofusarin BGC genes) with a concomitant decrease in DON production (and decreased *Tri6* and *Tri5* gene transcription) (Reyes-Dominguez et al., 2012). Although the exact mechanism for which *Hep1* and *DIM-2* exert control upon the regulation of aurofusarin and trichothecene BGC transcription is unclear, it is apparent that both the expression of aurofusarin and trichothecene BGCs are inversely linked in *F. graminearum* Δ*Hep1* and Δ*FgDim-2*/Δ*FgRid* mutants and that DNA methylation play a role in influencing BGC gene expression.

Under NPN conditions, both WT and Δ*FgDim-2*/Δ*FgRid* mutant strains were observed to produce trichothecenes, fusaoctaxins, and gramillins and by day 14, growth of the WT strain progressed into advanced stressed conditions, conducive to produce conidia that coincided with the production of the molecule FARI (Fusarium Asexual Reproduction Inducer—a universal regulator for asexual spore formation in *Fusarium*) (Qi et al., 2018) and pigment molecules such as aurofusarin, fucofusarin, and rubrofusarin. FARI is a stereospecific signaling molecule that initiates a signaling cascade involved with Gpmk1 MAPK and *Fg*LaeA-*Fg*VeA velvet pathways (Qi et al., 2018). The *Fg*LaeA carries a conserved methyltransferase domain and is proposed to play a role in chromatin-based regulation of asexual development (in the dark) in *F. graminearum*, along with secondary metabolism and regulatory pathways essential for disease development in wheat (Kim et al., 2013). Loss of function of *Fg*LaeA—*Fg*VeA complex has been associated with a significant reduction in asexual sporulation and the reduction/abolishment of aurofusarin (and associated pigments) in *F. graminearum* (Jiang et al., 2011; Lee et al., 2012; Kim et al., 2013). In our study, loss of DNA methylation in Δ*FgDim2*/Δ*FgRid* mutant strains caused a failure to transition toward a metabolic state associated with asexual sporulation (by 14 days growth); rather a continued production of trichothecenes, fusaoctaxins, and gramillins (initial metabolic state) was observed, as evidenced by a relative increase in signal intensity compared to WT controls.

Under NPN condition, *F. graminearum* is triggered to produce trichothecenes and the transcription factor TRI6 associated with the trichothecenes gene cluster acts as a global regulator of secondary metabolism triggering the concomitant production of fusaoctaxins and gramillins (Shostak et al., 2020). All three classes of metabolites are reported virulence factors in plants. The metabolite 15-ADON is required for kernel to kernel spread of the *F. graminearum* through the rachis in wheat heads; Fusaoctaxins act to prevent callose deposition in plant cell walls and allows for cell to cell proliferation of hyphae; Gramillins exhibit a phytotoxic effect upon corn silks and during leaf infiltration in maize (Jansen et al., 2005; Bahadoor et al., 2018; Jia et al., 2019). Sustained production of gramillins in the Δ*FgDim2*/Δ*FgRid* mutant strain under NPN conditions resulted in the identification and characterization of new gramillin molecules with structural variations due to the incorporation of different amino acid residues. Although production of 15-ADON was observed to be elevated in Δ*FgDim2*/Δ*FgRid* strain compared to WT in NPN conditions, pathology tests showed a reduction in virulence with the Δ*FgDim2*/Δ*FgRid* mutant strain. A similar phenomenon was observed with the NADPH oxidase mutant *Noxa/b*, where the strain was compromised in virulence but had the full potential to produce 15-ADON in culture (Wang et al., 2014). It is conceivable that DNA methylation might play a role in transitioning between growth/metabolic states during host plant infection and the loss of an ability to regulate metabolic change during infection may explain the discrepancy between the ability to produce 15-ADON and virulence.

Among genomic features, methylation density was greatest in repetitive elements, in accordance with studies that suggest DNA methylation plays a critical role in the silencing of these elements (Deniz et al., 2019). Both inter-and intragenic methylation were also observed, but it remains unclear which features are critical to gene regulation by methylation. Elevated gene body (intron/exon) methylation in PN conditions may be important to maintain homeostasis, while decreased gene body methylation may allow for more rapid changes to gene expression under NPN conditions (Figure 3). Such gene body methylation is still poorly understood in fungi, however, a study in *Candida albicans* suggested that DNA methylation is predominantly located in the gene body and modulates transcriptional activity by acting as a repressive mark (Mishra et al., 2011). A majority of methylated genes in *C. albicans* encoded proteins involved in environmentally cued pathways, suggesting gene body methylation plays an important role in the regulation of transcription under changing environmental conditions (Mishra et al., 2011) Similarly, in *Ganoderma sinense*, transcriptional repression of BGC was linked to DNA methylation in their gene bodies (Zhu et al., 2015). The methylation density in *G. sinense* was similar to *F. graminearum* at 1.8%. Taken together, these results suggest a diverse role for gene body DNA methylation in fungi, and hint at a function in environmental sensing and trigger for secondary metabolite production.

## Data Availability Statement

The datasets presented in this study can be found in online repositories. The names of the repository/repositories and accession number(s) can be found below: NCBI BioProject [accession: PRJNA587083].

## Author Contributions

CB, RS, and DO: conceived and designed the experiments. CB and AS: performed the experiments. CB, AS, DO, and RS: analyzed the data. CB and RS: wrote the paper. CB, RS, DO, and OR: revised the paper. All authors contributed to the article and approved the submitted version.

## Conflict of Interest

The authors declare that the research was conducted in the absence of any commercial or financial relationships that could be construed as a potential conflict of interest.

## Supplementary Material

Supplementary Figure 1 **(A)** *F. graminearum* DNA methyltransferases FGSG_10766 and FGSG_08648 are evolutionarily similar to DNMTs, *Nt*DIM-2, and *Nt*RID from *Neurospora tetrasperma*, respectively. Protein sequences from 35 eukaryotic DNA methyltransferases were used to infer phylogenetic relationships in CLC Genomics Workbench under a maximum-likelihood model. A prokaryote (*E. coli*) was used as an outgroup. **(B)** Protein sequences from 35 eukaryotic DNA methyltransferases.

Supplementary Figure 2 Schematic of *Agrobacterium tumefaciens* mediated transformation of *Fusarium graminearum* using USER Friendly Cloning. **(A)** A schematic of gene deletion of *FgDIM-2* construct and replacement with a hygromycin resistance marker. **(B)** A schematic of *FgDIM-2* complementation construct with the secondary resistance marker geneticin, G418. **(C)** A schematic of gene deletion construct of *FgRID* using G418 resistance marker. **(D)** A schematic of *FgRID* complementation construct with the secondary resistance marker hygromycin.

Supplementary Figure 3 Confirmation of *F. graminearum* mutant strains. **(A)** Relative expression of *FgDIM-2* and *FgRID* in mutant strains in NPN condition grown for 24 h. **(B)** Relative quantification of copy number of the DNMTs *FgDIM-2* and *FgRID* in all the strains. **(C)** Genomic sequence of the double mutant Δ*FgDim-2*/Δ*FgRid* strain aligned to the WT reference sequence in the gene regions of (1) *FgDIM-2* and (2) *FgRID*. **(D)** Confirmation of the mutant strains by PCR amplification with *FgDIM-2* and *FgRID* internal primers (I), hygromycin resistance internal primers (II), geneticin resistance internal primers (III), pGPD-*FgDNMT4* overlapping primers (IV), and positive control gene *Tri6* (V).

Supplementary Figure 4 Both *FgDIM2* and *FgRID* are required for perithecia production in *F. graminearum*. The images are representative of four technical replicates and between two-five biological replicates from the Wild-type, Δ*NoxA/B* (negative control), Δ*FgDim-2*, complemented strain Δ*FgDim-2/FgDIM-2*, Δ*FgRid*, the complemented strain *FgRid*: *FgRID*, and the double mutant strain Δ*FgDim-2*/Δ*FgRid*.

Supplementary Figure 5 Methylation density is identical in both the Watson and Crick DNA strands. DNA methylation sites were grouped into 10 kb bins for both the wild-type (WT), and the mutant (Δ*FgDim-2*/Δ*FgRid*) strains and the two environmental conditions. The average DNA methylation was plotted across the forward (red) and the reverse (blue) DNA strands. The average GC content (48%) is indicated in grey.

Supplementary Figure 6 A high-resolution differential methylation density map of regions 1 and 2 between *F*. *graminearum* wildtype (blue) and Δ*FgDim-2*/Δ*FgRid* strains (red) under **(A)** PN condition and **(B)** NPN conditions. The arrows represent the location of the regions in the *F. graminearum* genome.

Supplementary Figure 7 Validation of the RNA sequencing by RT-qPCR of two genes, FGSG_09595 and FGSG_02322 as assessed by standard curve and normalized against two reference genes, FGSG_16627 (GAPDH) and FGSG_09530 (βtubulin) using RNA extracted from mycelia grown for 24 h in the NPN conditions.

Supplementary Figure 8 Molecule structures of gramillins A, B, E, and F; amino acid residues are highlighted in color (HO-Gln, HO-glutamine; Ser, serine; DHA, dehydroalanine; Leu, leucine; Glu, glutamic acid; 2-AAA, 2- amino adipic acid; Cys, cysteine; 2-ADA, 2-amino decanoic acid). Two mass features, *m/z* 829.3613 [M+H]^+^ at RT 4.40 and *m/z* 843.3755 [M+H]^+^ at RT 4.43 were observed in the RAW data. Their molecular formulas were predicted using MassWorks^TM^ software (v5.0.0, Cerno Bioscience) yielding formulas of C_35_H_56_N_8_O_11_S_2_ (Δppm = 2.204, spectral accuracy 92.665%) and C_36_H_58_N_8_O_11_S_2_ (Δppm = −0.738, spectral accuracy 93.677%), respectively. MS^2^ and MS^3^ data revealed fragmentation patterns nearly identical to the gramillin A and B (Bahadoor et al., 2018). This, together with their predicted molecular formulas, suggested their structures may reflect a difference of a loss of H_2_O from gramillins A and B, respectively. Elucidation of the MS^2^ and MS^3^ fragments for both molecules, (1) with corresponding *m/z* 829.3613 [M+H]^+^ and (2) with corresponding *m/z* 843.3755 [M+H]^+^, revealed two new gramillin analogs, gramillin E (1) and gramillin F (2). The structures of gramillin E and F are identical to that of gramillin A and B, respectively, with the exception of a dehydroalanine residue incorporated in place of serine.

Supplementary Table 1 DNMTs from *F. graminearum* share identity with RID2 and DIM-2 from *Neurospora tetrasperma*. Protein sequences were obtained from Uniprot (https://www.uniprot.org/) under accession numbers Q8NJV8 (*NtRID*), G4UYB9 (*NtDIM-2*), I1RWH8 (FGSG_08648) and I1S1Y7 (FGSG_10766). The sequences were subjected to reciprocal protein-protein BLASTp searches on NCBI (https://blast.ncbi.nlm.nih.gov/Blast.cgi).

Supplementary Table 2 Whole genome bisulfite sequencing statistics for WT and Δ*FgDim-2*/Δ*FgRid* strains sequenced using Illumina HiSeq X PE150. Sequencing reads were deposited under accession # PRJNA587083 and mapped to the reference genome (Accession # SPRZ00000000) using CLC-Genomics Workbench.

Supplementary Table 3 DNA methylation is present under all three cytosine contexts, CpG, CHG and CHH in WT and Δ*FgDim-2*/Δ*FgRid* strains, under both 24 h PN and 6 h NPN environmental conditions. Methylation level remained consistent between strains and environmental conditions. Methylation level is defined as the proportion of methylation at any given site in a population.

Supplementary Table 4 DNA methylation exists in WT and Δ*FgDim-2*/Δ*FgRid* strains with minor differences between strains at the genome wide level. DNA methylation density was defined as the number of methylated cytosine as a percentage of the total cytosine. DNA methylation was predominantly identified in the asymmetrical CHH context in both strains.

Supplementary Table 5 Primers used in this study.

Supplementary Data Sheet 1 Summary and classification of genes identified in Region 1 in the WT strain between PN and NPN conditions.

Supplementary Data Sheet 2 Repetative elements identified in the Fusarium graminearum genome using RepeatModeler (v1.0.11). Default output is Stockholm format.

Supplementary Data Sheet 3 Identity of genes in the SMCs expressed in NPN environmental condition.

Supplementary Data Sheet 4 Mass features identified by UPLC-MS with significant changes in abundance between the WT and mutant strain.

Supplementary Data Sheet 5 Contains all the Supplementary Figures.

Supplementary Data Sheet 6 Contains all the Supplementary Tables.
