# Supplementary material for: DNA Methylation Is Responsive to the Environment and Regulates the Expression of Biosynthetic Gene Clusters, Metabolite Production, and Virulence in Fusarium graminearum
Source: Front Fungal Biol. 2021 Jan 15;1:614633. doi: 10.3389/ffunb.2020.614633 (PMC10512235; doi:10.3389/ffunb.2020.614633)
Supplement: Supplementary Figure 1 — (A) F. graminearum DNA methyltransferases FGSG_10766 and FGSG_08648 are evolutionarily similar to DNMTs, NtDIM-2, and NtRID from Neurospora tetrasperma, respectively. Protein sequences from 35 eukaryotic DNA methyltransferases were used to infer phylogenetic relationships in CLC Genomics Workbench under a maximum-likelihood model. A prokaryote (E. coli) was used as an outgroup. (B) Protein sequences from 35 eukaryotic DNA methyltransferases. [file Data_Sheet_5.pdf]

Figure S1A

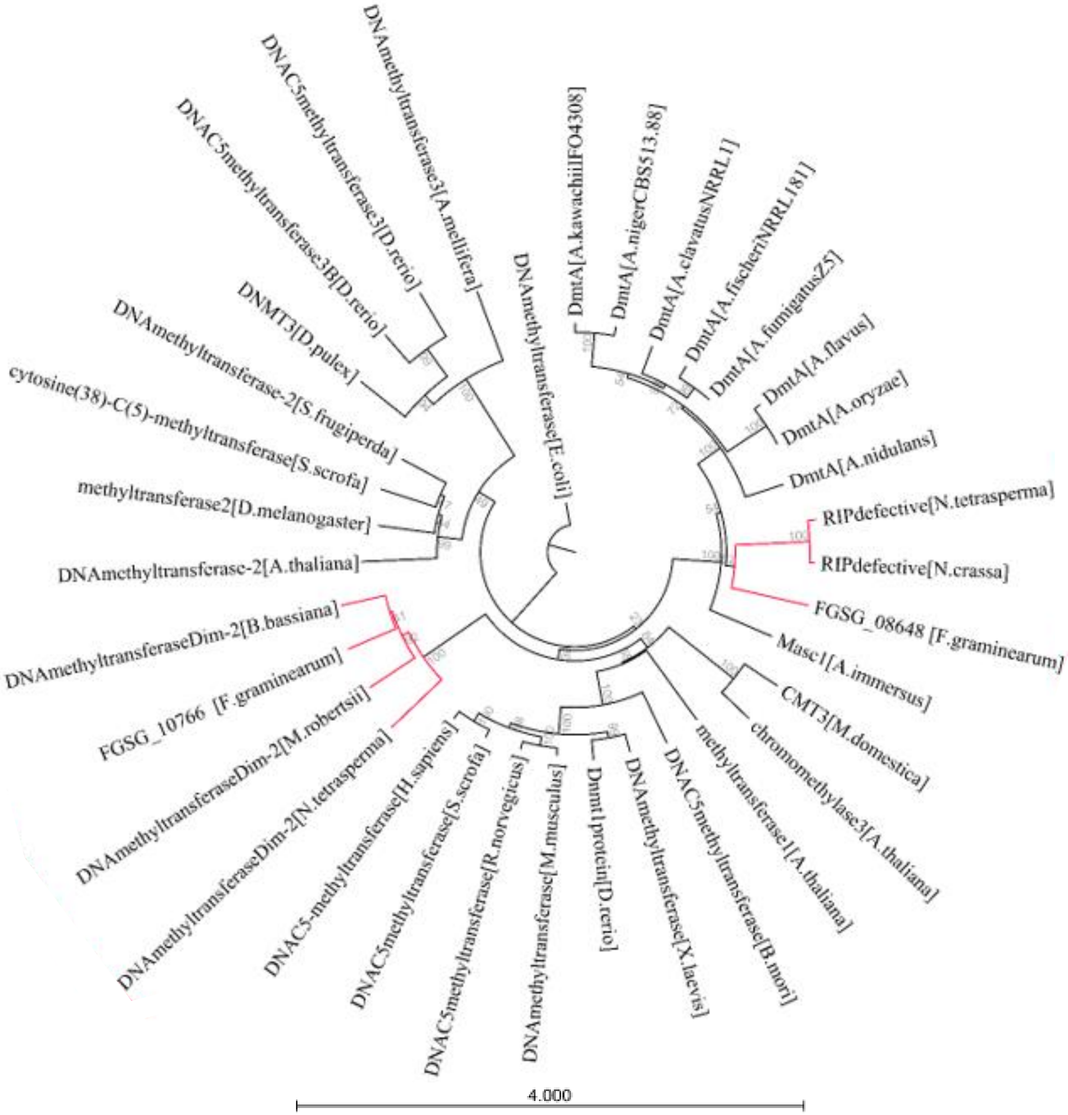
DNAmethyltransferase3[A.mellifera]
DNAC5methyltransferase3[D.rerio]
DNAC5methyltransferase3B[D.rerio]
DNMT3[D.pulex]
DNAmethyltransferase-2[S.frugiperda]
cytosine(38)-C(5)-methyltransferase[S.scrofa]
methyltransferase2[D.melanogaster]
DNAmethyltransferase-2[A.thaliana]
DNAmethyltransferaseDim-2[B.bassiana]
FGSG_10766 [F.graminearum]
DNAmethyltransferaseDim-2[M.robertsii]
DNAmethyltransferaseDim-2[N.tetrasperma]
DNAC5-methyltransferase[H.sapiens]
DNAC5methyltransferase[S.scrofa]
DNAC5methyltransferase[R.norvegicus]
DNAmethyltransferase[M.musculus]
Dnmt1protein[D.rerio]
DNAmethyltransferase[X.laevis]
DNAC5methyltransferase[B.mori]
methyltransferase1[A.thaliana]
chromomethylase3[A.thaliana]
CMT3[M.domestica]
Masc1[A.immersus]
FGSG_08648 [F.graminearum]
RIPdefective[N.crassa]
RIPdefective[N.tetrasperma]
DmtA[A.nidulans]
DmtA[A.oryzae]
DmtA[A.flavus]
DmtA[A.fumigatusZ5]
DmtA[A.fischeriNRRL181]
DmtA[A.clavatusNRRL1]
DmtA[A.nigerCBS513.88]
DmtA[A.kawachiiIFO4308]
DNAmethyltransferase[E.coli]
4.000

Figure S1B

```
>EZH35037.1 DNA methyltransferase, partial [Escherichia coli O26:H11 str. 2010C-3871]
ECYRDNFSHPIKKLDLSNVDDVVRELKDIDFDMIIGGPPCQDFSHAGLRIEGARANLTRSFSEIIKRIKP
KWFVMENVDRALRSGAYLEARGIFKESGYGLTEIVLDASKCGVPQKRKRLFVIGKLDVRDGFILNEVMCG
ISKDSMTVRNYLGDSLGIEYYYRHPRNYNRRAIFSIDEPAPTVRGVNRPIPDGYLGHA

>XP_001823049.2 C-5 cytosine methyltransferase DmtA [Aspergillus oryzae RIB40]
MSSIQAREPSILEIADPRPTTLEVYDSDTDSSVTLDHDSDGPDYADDVVVISDNRPRDPTPFLLSTNAAA
NLRELVDLTLPTFQAFDEDDYCTDEEFELLLQSWQLHTPSPRVTTPVDETRATTVLPVEEVCIDGILYKP
GQSLELYDGSYLRICTVLKDSMDAVSFSGRRLLKTRNHAGTYVPKERNELVWIANATEIIPFQMAKKFVS
MNFTNICAIRGDPQKLVNPNHLWCRLKETLEDGEVSIEYVSFVEADEGHKIEPAILRQLWRGKSRAFGEE
DRPRESSPVIVLDNPDPVIDLTCSESVDEQKRRRQYTFGDGFCGAGGVSCGARRAGLYNKWAFDNSEHAT
STYRLNFEHAYCELSDIFSFLTSNDEFLRVDVSHSSPPCQTWSSAHTIEGANDDANSACVFSSADLIRRA
KPRVHTMEETNGLLDRHRDTLHRVINDFIEIGYSVRWGILRLLEYGVPQTRKRLLVIASGPGETLPPFPR
PTHGPGLGEYPTISQAIQNIPLGAPDHDVQAALSRGMDKPPYDPHRPAGTITCGGGDNNYHPSGRRNFTN
RELACLQTFPRNFRFGRRQVRKQIGNAVPPLLAEAMYREIIRTLKKTDEDEASR


>XP_001396716.1 C-5 cytosine methyltransferase DmtA [Aspergillus niger CBS 513.88]
MAPFSFSMPHLCESDNDWPSDLMSIDESSDHSSVTVENDPDRVLNIQTGIRSGPLFENNPTPAEFDEIIE
LDSDTEEGFNEQDYLTDAAFNRLLQNWHRAPQGQDADAEVQFIDVQPAKRPLTEWCANGIVYTPGQSLEI
FDGNFIRIESISQHVVSKEIFFNGRHLLRATNHKGTYIPKWPNELIWIANEKRNIGLPLIKRIVNITFTN
TCHVEHDPRKKQRPHGLFCRLKENYRLKPDDPASVEYISFEEADEGYRAHPSSLRKAWRGTTRPFGASDA
PQAIDLEGPVIDLTGQSTDDKSSRQYTFGDGFCGAGGVSCGALKAGLRPTWAFDNSRHAINTYRLNFRDA
ECEDSDVFTFLTNDYAFLKVDVTHGSPPCQTFSPAKTIQCATDDANSACIFSCAEMIRKAKPRVHTMEET
SGLYERHRETFHRVVQDFIEIGYSVRWTILKCADYGVPQLRRRLVIIASGPGETLPPFPEATHGLPGSGL
RDYVTINQVISRIPRGAPDHDVEGALQRSAIDRRPFNANRQARTITCGGGENYHPSGRRGFTNREFACLQ
TFPMRFRFGPREVRKQIGNAVPPKLAEAIYRSVKASLQRTDEEETNLQRRGLH


>AAO37378.1 c5 cytosine methyltransferase DmtA [Aspergillus nidulans]
MRQSAYIYLTFVSSCTVCELLLYTFLTRLSLPSCSRDMHNFYRPSVIVDDASDASSVTIDHDPDRLYFLR
EDSDTATREFIDLTEDETTHDGEYITDECFEVLREDWLASACASSPLSPELPEGQLLEEVCDADGIVYKP
GNSVELHDGAFFRICSIRQALKGNIILTGRRLLKFKDHPDKYLPQWRNELIWVADETAEIPLWFVRRFVS
VHFSNVCPIGQDCQKNNNPDGLFCRLKRVIQKETTSIEYLTFEEADAGFRAPSASLRHGWRGETAPFGSK
EEAETPVIVLDDDNDFQDTILKQKAQRKYTFGDGFCGAGGVSCGAEAAGLDIKWAFDLCPHAAATYRLNF
PNVECEGSDIFSFMTSNEEFMRVDISHGSPPCQTFSPAHTIPGPNDDANSAAIFSCWDLIRKAKPRVHTM
```

EETSGLFDRHQQVFLRVICDFIETGYSVRWALLNCMWYGVPQSRKRLIIIASGPGESLPRLPRPTHGLPG
SGLRDLTTISQAIRDIPTGSPDHDVVAARGRGVHNRRAPFDGNRQARTITCGGGDNYHPSGLRGFTLREF
ACLQTFPLGFRFLGGRTQVKRQIGNAVPPLLAKAVFKEIIRSLQDTDERELHENQ

>GAA90610.1 C-5 cytosine methyltransferase DmtA [Aspergillus kawachii IFO 4308]
MAPFSFAIPHLCESDDDWPSDAMSIDGDSDRSSVTVENDPDLVLNIQTGYRSGPATERDSSPAEFNEIIE
LDSDTEEGFNEKDYLTDAAFNRLLQDWNKAPRRQDVDEDVQFIDVAPAKRPLKEWCANGIVYTPGQSLEI
FDGRFIRIESISQHVVSKEIFFTGRHLVRATGHKGTYIPKWPNELIWIANDTRNIGLPLIKRIVNVIFTN
ACHVEHDLLKKRRPHALFCRLKENFRDKPDDPASVEYLSFDEADEGYRTYPSSLRKAWRGTTRSFGASDA
PQAIDLEGPVIDLTEQSTENKSSRQYTFGDGFCGAGGVSCGARKAGLRPTWAFDNSRHAVNTYSLNFRDA
ECEESDVFSFLTNNYDFLKVDVTHGSPPCQTFSPAKTVQCATDDANSACVFACAELIRKAKPRVHTMEET
SGLYERHRETFHRVVLDFIEIGYSVRWTILNCADFGVPQLRRRLVIIASGPGETLPPFPEATHGLPGSGL
RDYVTINQVISRIPRGSSDHDVEGALQRSAIARRPFDANRQARTITCGGGENYHPSGRRGFTNREFACLQ
TFPMRFRFGPREVRKQIGNAVPPKLAEAIYRSVKASLQRTDEEETNPERRGPF

>KMK55222.1 C-5 cytosine methyltransferase DmtA [Aspergillus fumigatus Z5]
MSLRHSWRGETRPFGSADANSQPQSPIIVLDADPVIDLTGIEKEDVRVQKKHRKYTFGDGFCGAGGVSCG
ASKAGLHIKWAFDKSENAITTYRLNFATAVCEACDIFCFLTNKPEELKVDVSHGSPPCQTFSPAHTINSV
NDDDNSACIFSCADMIKKSRPRVHTMEETSGLFDRHKETFHRVIQDFIEIGYSVRWRILNCMDYGVPQSR
RRLIIIASGPGEVLPPFPKPTHGLPGSGLSDYTTINQMIANIPPNAPDHDIEGARSRGLRNGTRVPFDPN
QQAKTVTCSGGENNYHPSGTRGFTNREFACLQTFPLDYRFGAREVRRQIGNAVPPALSKAIYREIIKSLQ
RTDEQELRG

>XP_001257580.1 C-5 cytosine methyltransferase DmtA [Aspergillus fischeri NRRL 181]
MNRPTNDSLHSQELPQPSEPICLDDSDDDSSVCDTESNTDSSVTIDNDPDRSFFLDDEQFHPGGSRRTPT
PFHIGPQTHPEIIDLTSGPELLVTEGDYLTDECFERLLRDWGRPGPAAASLPIQEASEWEKRPIDHACVD
GIVYKAGHSLELHNGLYLRIETVLQDTAEEVFFRGRHLIRTRHHKGTYIPKWSNELVWIVNESTEVPLSF
VKRFINIRFTSCCHVEQDLQKRHRPNDLFCRLKENLDSSQAAVEYLSFEESDEGFAIDSTSLRQSWRGET
RPFGSADANPQPQSPVIVLDADPVIDLTEIEKEDARVQKKHRKYTFGDGFCGAGGVSCGASKAGLHIKWA
FDKSENAITTYRLNFATAVCEACDIFCFLTNKPEDLKVDVSHGSPPCQTFSPAHTINSVNDDDNSACIFS
CADMIKRSRPRVHTMEETSGLFDRHKETFHRVIQDFIEIGYSVRWRILNCMDYGVPQSRRRLIIIASGPG
EVLPPFPKPTHGLPGSGLSDYTTINQMIANIPPDAPDHDIEGARSRGLRNGTRVPFDPNQQAKTVTCSGG
ENNYHPSGTRGFTNREFACLQTFPLDYRFGAREVRRQIGNAVPPALSKAIYREIIKSLQRTDEQEFRVGD
SW

>EAW08753.1 C-5 cytosine methyltransferase DmtA [Aspergillus clavatus NRRL 1]
MNQTSCIEHSDTVVVDDDESDTASSVTIDNDPDRSFFLDETRPGTRLRTPTPFRLGPHTHQEIIDLTSGP
ELLVTDGDYLTDECLERLLHDWEHPEAAGAAPPARSDRPLVRTEILRRHPIEHACVDDIVYKPGQSLELE
KGKYLRITTILQDADGNVFFQGRHLISTKNHKESYMPKWSNELVWILNETSEIPLAAATRFVNIHFTNCC
HVDHDFQKKSQPFDLFCRLKENLDPTKASVEYLSFEDSDEKFRIESATLRESWRGETRPFGNAKRDISRS
PVITLDGVEPIIDLTSQNQSISDQKMRRQYTFGDGFCGAGGVSSGAQKAGLHIKWAFDQSEHAIATYRMN
FETALCEQSDIFSFLTNGPDFLKVDVSHGSPPCQTFSPAHTINSANDDANSACIFSCADMIQHCKPRVHT
MEETSGLYDRHQETCYRVILDFIEAGYSVRWKKLNCMDYGVPQPRKRLVIIASGPGEVLPPFPRPTHGLP
GSGLLPYATINSVISNIPPNALDHDIEGARIRGRRNGLRAPFSPNQQAKTITCSGGENNYHPSGTRGFTN
REFASLQTFPLEHLFGTTNVRRQIGNAVPPALAWAVYREIINSLHRTDEEEIRSRSM

>EED51371.1 C-5 cytosine methyltransferase DmtA [Aspergillus flavus NRRL3357]
MSSIQAREPSILEIADPRPTTLEVYDSDTDSSVTLDHDSDGPDYADDVVVISDNRPRDPTPFLLSANAAA
NLRELVDLTLPTFQAFDEDDYCTDEEFELLLQSWQLHTPSPRVTTPVDETRATTVLPVEESLELYDGSYL
RICTVLKDSMDAVSFSGRRLLKTRNHAGTYVPKERNELVWIANATEIIPFQMAKKFVSMNFTNICAIRGD
PQKLVNPNHLWCRLKETLEDGEVSIEYVSFVEADEGHKIEPAILRQLWRGKSRAFGEEDRPRESSPVIVL
DNPDPVIDLTCSESVDEQKRRRQYTFGDGFCGAGGVSCGARRAGLYNKWAFDNSEHATSTYRLNFEHAYC
ELSDIFSFLTSNDEFLRVDVSHSSPPCQTWSSAHTIEGANDDANSACVFSSADLIRRAKPRVHTMEETNG
LLDRHRDTLHRVINDFIEIGYSVRWGILRLLEYGVPQTRKRLLVIASG

>XP_007820646.1 DNA methyltransferase Dim-2 [Metarhizium robertsii ARSEF 23]
MRSELEASETEHDTPVLKETLDTEAVWACENDSASCQDDLSQWSETLRASSSSPSVFSEESSNLAVEIPD
FQLSSPRSCYEPFHPPAPPKSEEDALECFGLSVQRQSSPSSGHFELQLDEFAVYLDTENYPCEMRCLHHL
YARPGYSNFFFDGILSNNGQGKTFMKRIPISAVPVGQYGLQYHSVGNQIWLQSTYCDQSDIFYKLCLPAR
EYRRFFKAFLWVANLAKHFVDFLVFMEEKGTRVTIRHFQSAFGDWLLVVHGDAPDLMDWLAQHASSDFRT
SVNANISFLYKEYRGIITDREYGYHDIWAEIWDFTKYPDLSTGTAQELTVVTDYVYHRFSDLPFGNRLHN
FPLSRRASELRERASLKHGLELACASHGPATSSFVNKTGGDISDITRAIKPGDTISTRRDGEVSRSMRRR
ELSNDFAGVDRRFALVQKVCERSDGGRYFEVLWYYRPSDTLCGLMKYPWGNELFLSDHCSCSEENKIQDC
EVLRVHSVQFKGDSTTTSEFFCRQRYLSKEKKWIALDDSDLICSHLNPRLQDSTSGKYRPGDTFLIHVNV
ASSTSEACELISVNTRGAGTRLCFRKLLQRSNVDSTAVTPARNELVYSNESIECDPSGIVGKCHVRFFAH
DENIPAPYNRAGVGGYFYVTHRVSPAGLCVPLRSVPSSLRQGFDPAVVISELRGLDLFCGGGNFGRGLEE
GGAVRMSWANDCNARAIHTYMANCSHPNLMSPFLGSIDDFQREAFSGNFSKSVPTIGSVDFISGGSPCPG
FSTLTNDRTTDEQRKNQSLVAAFASCVDLYRPKYGLLENVPGILPSKANREQDVLGQFMCAIVGIGYQAQ
VFYLDASSCGSAQRRSRVFISFAAPNYKLPGKPQQTHSHPPDTRYTSLGKLPTRRHVLAERDFLRATPFS
YSVARQVSSGLPKIHNGQTDTCIAFPDHRLARGLTKGLKARISLIPNRPWGMNLRSASRGILTPAERGVF
VTINRKTSKPNTEPHKLDRQSSAYGRLCPNQLIGAITTIQVPNDRKHGRQLHWHENRGLSVMEARRAQGF
LDDEVILGTPAEQYKIVGNSVAREVALALGLSIREAWAESCADDQDTEAAKPLSSSLARGALGGNADHGP

RHTSAAEASIRTRVIPQKRRRRATSRMLVVTGRAKEKCPPECIDAR

>XP_008598218.1 DNA methyltransferase Dim-2 [Beauveria bassiana ARSEF 2860]
MKLTADLDLDLDLNLDLDLDLDLDAISSEQHDTRILATTCLPGVLSGIDYDGSYSSHGSKDDDLEEDGPT
GSYPISVHIPECMLISPRSCYTPFDPPCPVASERDALRILLPQRYAKNGFLELKLDDFAVYCDTKFRPEE
MRSLSQLGTEPHSTPLFFDGMLSHGTQSLYVRRVRIHALPIGNYGQESRHQTRDNIWIQSEENATSDVFY
KLGSPAPEYKRFFELFVWVADLAKYFVDFLTVMRAIERPVTIHHFRRDFAAWLLALYAKSDKIEAVKTWM
CKHPRQDYRSAVNANVDFLHKESVGVLGERRAYFHDLWNEVFFFQTYTPYIAHNEDKCTIVTKYIYDCFH
HLPFGKRLKVVDLSAHTKRLRLEVIAQRPMLPINGTQVTPAGHGKIGPGDTISTARDTVDSGTDWEKEEA
KGDEYSDLWFALVQSVSVNSDGVRVFEVIWYYRPVDTLCGLMQYPWSDELFLSDHCSCEEGTKIREDEVR
SVHEVDFGGTPETTKEFFCRQSYLSDDKIWVTLTQSHLTCRHNNDAPIPDSAEEYCLGDTVLVKSGRNSN
VCHPCEIIETKQENGRLRFVFRKLPIRHAVDLTARNAPPNEVVYTDEIVKSGPSSIVSRCHVRWFPQTRP
VPTPYDRDGVGAFFFLTHQKKWTGNGYITSPLEEAPPSLNQGHDPQQDSKPKLRGLDLFCGGGNFGRGLE
EGGSVTMKWANDVNSKALHTYMANTEPGQIYPFLGSIDDFQRLAFQGRFADNIPQVGEVDFISGGSPCPG
FSQLTNDKETDAQRKNQSLVAAFASCVDLYRPKYGILENVAGIIQKHQNRGHDVFSQLMCALVGMGYQAR
LVLLDALSCGSAQVRSRVFIIFAAPGWTLPEAPIQTHSHQPNVKNFKLGRLPTGEPMARREVASCTPFSY
KSASQAAAGLPSLYDATPDICIAFPDHRVVSHTNTNTMRNRIRLIPKAPYGMNFSQAWYGRLGTRPRVAG
RGVLTRSERNMFSLRGEATMSTSIQSRAYGRQPPNQPMATIVTRASPRDAKQGRTIHWSEDRCLSVMEAK
RGQGFLDEELLLGSADVQYRIIGNSVAREVSLALGVVFAEAVMRSYGQGVEDLAVDSEDEVTIKTEMSVP
TTPGATRQQGENNGRNKRRRVG

>EGZ69028.1 DNA methyltransferase Dim-2 [Neurospora tetrasperma FGSC 2509]
MDLPDRSHGGMFIDVPAETMGFQEDYLDMFASVLSQGLAKEGDYVHHQPLPAAKEECLEPIAATTITPSP
DDPQLQLQLELEQQFQTESGLNGLDPAPAPESEDEADLPDGFSDESPDDDFVVQRTERVAVEDLLKAAKA
AGKNKEDYIEFELHDFNFYVNYAYHPQEMRPIQLVATKVLHDKYYFDGVLKYGNTKHYVTGMQVLELPVG
NYGASLHSVKGQIWVRSKHNAKKEIYYLLKKPAFEYTRYYQPFLWIADLGKHVVDYCTRMVEKKREVTLG
CFKSEFIQWASKAHGKSKAFQNWRAQHPRDDFRTSVAANIGYIWKEINGVAGAKRAAGDRLFRELMIVKP
GQYFRQEVPPGPLVTEGDRTVAATIVTPYIKECFGHMILGKVLRLAGEDAEKEKAVKLAKRLKIENKNDT
KADTKDDIMNDTATESLPTSLRALPVQVLEATPIESDIVSIVSSDLPPSENNPPPLTNGSVKPKAKANPK
PKPSTQPLHAAHVKYLSQELVNKIKVGDVISTPRDDSSNTDTKWKPTDTDDHRWFGLVQRVHTAKTKSGR
GLSSKSFDVIWFYRPEDSPCCAMKYKWRNELFLSNHCTCQEGHYARVKGNEVLAVHPVDWFSTPESNKGE
FFVRQLYESEQRRWITLQKDHLTCYHNQPPKPPTTPYKPGDTVLATLSPSDKFSDPYEVVEYFTQGEKET
AFVRLKKLLRRRKVDRQDAPANELVYTEDLVDVRAERIVGKCVVRCFRPDERIPSPYDRGGTGNIFFITH
RQDHAGCVPLDTFPPTLRQGFNPLGNLGKPKLRGMDLYCGGGNFGRGLEEGGVVEMRWANDIWDKAIHTY
MANTPDPNKTHPFLGSVDDLLRLALEGKFSDNVPRPGEVDFIAAGSPCPGFSLLTQDKKVLNQVKNQSLV
ASFASFVDFYRPKYGVLENVSGIVQTFVNRKQDVLSQLFCALVGMGYQAQLILGDAWAHGAPQSRERVFL
YFAAPGLPLPDPPLPSHSHYRVKNRNIGFLCNGESYVQRSFIPTAFKFVSAGEGTADLPKVGDGKPDACV
PFPDHRLASGITPYIRAQYACIPTHPYGMNFIKAWNNGNGVMSKSDRDLFPSEGKTRTSDASVGWKRLNP

KTLFPTVTATSNPSDARMGPGLHWDEDRPYTVQEMRRAQGYLDEEVLVGRTTDQWKLVGNSVSRHMALAI
GLKFREAWLGTLYDESAAVATATATTTTAAAVGVTVPVMEEPRVGTTDSTRLSRSPVHTAVDLDDSKSER
SRSTTPATVLSTSSAAGDGSANAVGLEDNDNDNMEMMEVTRKRSSPAVDEEGMRPSKVQKVEVTVASPAS
RRSSRQTSRNPTASPLPEASKATTHEAPAPEEPESDAEYYSETYDKEGFDGDYHSGHEDQYSEEDEEEEY
AEPETMTVNGMTIVKL

>AAC49849.1 Masc1 [Ascobolus immersus]
MSERRYEAGMTVALHEGSFLKIQRVYIRQYHADNRREHMLVGPLFRRTKYLKALSKKVNEVAIVHESIHV
PVQDVIGVRELIITNRPFPECRKGDEHTGRLVCRWVYNLDERAKGREYKKQRYIRRITEAEADPEYRVED
RVLRRRWFQEGYIGDEISYKEHGNGDIVDIRSESPLQVLDGWGGDLVDLENGEETSIPGPCRSASSYGRL
MKPPLAQAADSNTSRKYTFGDTFCGGGGVSLGARQAGLEVKWAFDMNPNAGANYRRNFPNTDFFLAEAEQ
FIQLSVGISQHVDILHLSPPCQTFSRAHTIAGKNDENNEASFFAVVNLIKAVRPRLFTVEETDGIMDRQS
RQFIDTALMGITELGYSFRICVLNAIEYGVCQNRKRLIIIGAAPGEELPPFPLPTHQDFFSKDPRRDLLP
AVTLDDALSTITPESTDHHLNHVWQPAEWKTPYDAHRPFKNAIRAGGGEYDIYPDGRRKFTVRELACIQG
FPDEYEFVGTLTDKRRIIGNAVPPPLSAAIMSTLRQWMTEKDFERME

>AAM27408.1 RIP defective [Neurospora crassa]
MAEQNPFVIDDEDDVIQIHDEEEVEEEVAEVIDITEDDIEPSELDRAFGSRPKEETLPSLLLRDQGFIVR
PGMTVELKAPIGRFAISFVRVNSIVKVRQAHVNNVTIRGHGFTRAKEMNGMLPKQLNECCLVASVDTRDP
RPWREQAILDINPENVLTTRELRVTNAQFPRYRDKSADMAIKRQQVKDMGILVCRYSYVEYRHVDKPREW
SFIRVEENEADEGFRLSDDVLVNGWRGGKVPGGSFLPAGQEHGHGHVHNVDDLTSTLPSLTARGPKQVPS
DQKYTAGDTFAGAGGASRGITDAGVHLEFCVDNWEHAVASLNANFQGQDTTTYDIDMHNFIVNKEIRHRV
DILHLSPPCQVWSPAHTRPGQNDERNLAILFSCTHLIEKIRPRLFTVEQTFGILHPRLDNFFQSLVHGFT
DHGYSVRWKVVNFSHYGLPQPRRRLIMIGAGPGEKLPPFPSPTHGNGLKPVTTARQALAAIDGRRRYPLH
QPYLQPFPTRKAHWDGDKPLPYTVTCGAAENYHWSGLRQFTPQEYALLQGFPMHHKFAGNYIKKQIGNAF
PPIFVKLLYKHLVECLDKRDNIIRQAQARTEEAAPFQTPRKLVGSVGRNEVGSVGRNEEDDGEVTFLSSR
KRRRQFAAVEVIDNNNRISTHRSKRARRLVGNTPPCQQAAAAQAKQKTIIDLDEDISNLDLDQDRDDDRS
DTATIRESSVEVEASPRVSPVRRHPAAPPSSHLPLGPAGLTNPIQGGPSTSTSTRTGSKASSSSQQQTHH
NNMQGETVRRKLFFAVPPSRTEPFSSPSSTSSTSSSTTASSSAAGSSNGSNSSSPVVKKENQKGTRKEPM
ELFDD

>AAM27410.1 RIP defective [Neurospora tetrasperma]
MAEQNPFIIDDEDDVIQIHDEEEIEGEVAEVIDITENDIELSEPDRAFRSGPKEETLPSFSLRDQGFIVR
PGMTVELKAPIGRFAISFVRVNSIVRLRQAHVNNVSIRGHGFTRAKEMNGMLPKQLNECCLVASVDTRDP
RPWREQAILDINPENVLTTKELRVTNAPFPRYRDGFADTAIKRQQVKDMGILVSRYSYVEYHQTERPREW
SFVKMEEKEADEEFRLSDDVLVNGWRGGKVPGGSFLPAGQEHGHRHVHNVDDLTSTLPSRGTKQVPSDQK
YTAGDTFAGAGGASRGITDAGVHLEFCVDNWEHAVASLNANFQGQDTTIYDIDMHNFIVDKEIRHRVDIL

HLSPPCQVWSPAHTRPGQNDEKNLAILFSCTHLIEKIRPRLFTVEQTFGILHPRFDNFFQSLVHGFTDHG
YSVRWKVVNFSHYGLPQPGRRLIMIGAGPGEKLPPFPSPTHGNGLKPATTARQALAAIDERRRYPLHQPY
LQPFPTRKAPWDGDKPLPYTVTCGAAENYHWSGLRQFTLQEYALLQGFPMHHKFAGSYIKKQIGNAFPPI
FVKLLYKHLVECLDKRDNIIRQAQARTEEAPFQTPRKLGNVGRNEEDDGEVTFLSSRKRRRQFAVVELID
NNNSNSSRSKRARLVGNTFPCQQPAAAQAKQKTVIDLDEDISNLDLDQDRDDGRSDTATIRESSVEVEVD
SPRVSPVRRHPAPPSHLLGPGPNPIRGGPSTCTRTGSKASSSQQQTHNMQGETVRRKLFFTAPPPRTEPF
SSPSSTSSTSATTTSSAESSNGSSSSSPVVKKENQKKGTSKEPMELFDDD

>NP_199727.1 methyltransferase 1 [Arabidopsis thaliana]
MVENGAKAAKRKKRPLPEIQEVEDVPRTRRPRRAAACTSFKEKSIRVCEKSATIEVKKQQIVEEEFLALR
LTALETDVEDRPTRRLNDFVLFDSDGVPQPLEMLEIHDIFVSGAILPSDVCTDKEKEKGVRCTSFGRVEH
WSISGYEDGSPVIWISTELADYDCRKPAASYRKVYDYFYEKARASVAVYKKLSKSSGGDPDIGLEELLAA
VVRSMSSGSKYFSSGAAIIDFVISQGDFIYNQLAGLDETAKKHESSYVEIPVLVALREKSSKIDKPLQRE
RNPSNGVRIKEVSQVAESEALTSDQLVDGTDDDRRYAILLQDEENRKSMQQPRKNSSSGSASNMFYIKIN
EDEIANDYPLPSYYKTSEEETDELILYDASYEVQSEHLPHRMLHNWALYNSDLRFISLELLPMKQCDDID
VNIFGSGVVTDDNGSWISLNDPDSGSQSHDPDGMCIFLSQIKEWMIEFGSDDIISISIRTDVAWYRLGKP
SKLYAPWWKPVLKTARVGISILTFLRVESRVARLSFADVTKRLSGLQANDKAYISSDPLAVERYLVVHGQ
IILQLFAVYPDDNVKRCPFVVGLASKLEDRHHTKWIIKKKKISLKELNLNPRAGMAPVASKRKAMQATTT
RLVNRIWGEFYSNYSPEDPLQATAAENGEDEVEEEGGNGEEEVEEEGENGLTEDTVPEPVEVQKPHTPKK
IRGSSGKREIKWDGESLGKTSAGEPLYQQALVGGEMVAVGGAVTLEVDDPDEMPAIYFVEYMFESTDHCK
MLHGRFLQRGSMTVLGNAANERELFLTNECMTTQLKDIKGVASFEIRSRPWGHQYRKKNITADKLDWARA
LERKVKDLPTEYYCKSLYSPERGGFFSLPLSDIGRSSGFCTSCKIREDEEKRSTIKLNVSKTGFFINGIE
YSVEDFVYVNPDSIGGLKEGSKTSFKSGRNIGLRAYVVCQLLEIVPKESRKADLGSFDVKVRRFYRPEDV
SAEKAYASDIQELYFSQDTVVLPPGALEGKCEVRKKSDMPLSREYPISDHIFFCDLFFDTSKGSLKQLPA
NMKPKFSTIKDDTLLRKKKGKGVESEIESEIVKPVEPPKEIRLATLDIFAGCGGLSHGLKKAGVSDAKWA
IEYEEPAGQAFKQNHPESTVFVDNCNVILRAIMEKGGDQDDCVSTTEANELAAKLTEEQKSTLPLPGQVD
FINGGPPCQGFSGMNRFNQSSWSKVQCEMILAFLSFADYFRPRYFLLENVRTFVSFNKGQTFQLTLASLL
EMGYQVRFGILEAGAYGVSQSRKRAFIWAAAPEEVLPEWPEPMHVFGVPKLKISLSQGLHYAAVRSTALG
APFRPITVRDTIGDLPSVENGDSRTNKEYKEVAVSWFQKEIRGNTIALTDHICKAMNELNLIRCKLIPTR
PGADWHDLPKRKVTLSDGRVEEMIPFCLPNTAERHNGWKGLYGRLDWQGNFPTSVTDPQPMGKVGMCFHP
EQHRILTVRECARSQGFPDSYEFAGNINHKHRQIGNAVPPPLAFALGRKLKEALHLKKSPQHQP

>AAI63894.1 Dnmt1 protein [Danio rerio]
MPTRTSLSLPEDVKERLQVLDEGGDSLSDEECVKEKLRLLQEFLLTDTQDQLKNLEDKLKSSELSTEVYM
SEVKAVLKKALGVGKEGDGVEQNGHSNGFSENGSHKDNGEQEGAMDTQDEGDTIKSPSAPKGRGGRRSKA
DSEPKKSPASSRVTRNTGKQQTIVSMFSRVPKRKSDELNGEPANGDTEIKTEETITEEVREEKRLKTEDE
KPEAENAANLKPVSTAKTPPPKCPDCRQYLDDSDLKFLQGDPDDALDEPEMLTDERLSLFDSNEDGFESY

EDLPQHKITNFSVYDKRGHLCPFDSGLIEKNVELYFSCAVKPIYDDNPCMDGGVPAKKLGPINAWWITGF
DGGEKALIGFTTAFADYILMDPSEEYSAIFALMQEKIYMSKIVVEFLQKNQDATYEDLLNKIETTVPPAG
LNFNRFTEDTLLRHAQFVVEQVESYDEAGDSDEQPIIITPCMRDLIKLAGVTLGKRRAARRQAVRHPTKI
EKDNKGPTKATTTKLVYLIFDTFFSDQIDQNNKDGGVKRQRCGVCEVCQAPDCGKCSACKDMIKFGGSGR
SKQACQKRRCPNLAVKEAEDDENMDEEDVLPVKDTKKMSQTKKKKQTKNKISWVGEPLKTEGKKEYYMKV
RVENEVLEVGDCVSVSPDDPSHPLYLARITALWDDGEKMFHAHWFCRGTDTVLGESSDPLELFLVDECED
MQLSFIHGKVNVFYKAPSENWYMEGGMDEDIKVIDDDGESFFYQLHYEGECARFETPPKVTPSEDCKYKF
CASCTRNEEREAESVPHAYEPLEDEESDSKVFYGLVNYKGEQYKVGDSVYLPPEAFNFVVKAASPVKRSH
RKDDVDEDLYPEYYRKSSDYIKGSNLDAPQPFRIGRIKEIFCNKRSNGKPDTTEIKLRLYKFYRPENTHK
GPKGAYHSDINQLYWSDEEATVSMTEVLTRCRVEYAEDLVESVQDYSNKGPDRFYFLEAYNAKTKSFEDP
PNHARSAVNKGKGKGKGKGKGKGKAAPQEPQDQEAQEPAVPKLRTLDVFSGCGGLSEGFHQAGISETHWA
IEMWDPAAQAFRLNNPGTTVFTEDCNVLLKLVMSGEKTNSLGQKLPQKGDVEMLCGGPPCQGFSGMNRFN
SRTYSKFKNSLVVSYLSYCDYYRPKFFLLENVRNFVSFKRSMVLKLTLRCLVRMGYQCTFGVLQAGQYGV
AQTRRRAIILAAAPGEKLPRYPEPLHVFAPRACSLSVAVDEKKYVSNVTRGNGGIYRTITVRDTMSDLPE
IRNGAAALEISYNGEPQSWFQRQIRGSQYQPILRDHICKDMSALVAARMRHIPLAPGSDWRDLPNIEVRL
RDGTTTKKLRYTHSDKKNGRSGTGALRGVCSCSEGKQCDPADRQFNTLIPWCLPHTGNRHNHWAGLYGRL
EWDGFFSTTVTNPEPMGKQGRVLHPEQHRVVSVRECARSQGFPDTYRFFGNVLDKHRQVGNAVPPPLSKA
IGLEVKKCVLEKMRENATEPVKQEKMELSD

>DNA (cytosine-5)-methyltransferase 1 isoform a [Homo sapiens]
MPARTAPARVPTLAVPAISLPDDVRRRLKDLERDSLTEKECVKEKLNLLHEFLQTEIKNQLCDLETKLRK
EELSEEGYLAKVKSLLNKDLSLENGAHAYNREVNGRLENGNQARSEARRVGMADANSPPKPLSKPRTPRR
SKSDGEAKRSRDPPASASQVTGIRAEPSPSPRITRKSTRQTTITSHFAKGPAKRKPQEESERAKSDESIK
EEDKDQDEKRRRVTSRERVARPLPAEEPERAKSGTRTEKEEERDEKEEKRLRSQTKEPTPKQKLKEEPDR
EARAGVQADEDEDGDEKDEKKHRSQPKDLAAKRRPEEKEPEKVNPQISDEKDEDEKEEKRRKTTPKEPTE
KKMARAKTVMNSKTHPPKCIQCGQYLDDPDLKYGQHPPDAVDEPQMLTNEKLSIFDANESGFESYEALPQ
HKLTCFSVYCKHGHLCPIDTGLIEKNIELFFSGSAKPIYDDDPSLEGGVNGKNLGPINEWWITGFDGGEK
ALIGFSTSFAEYILMDPSPEYAPIFGLMQEKIYISKIVVEFLQSNSDSTYEDLINKIETTVPPSGLNLNR
FTEDSLLRHAQFVVEQVESYDEAGDSDEQPIFLTPCMRDLIKLAGVTLGQRRAQARRQTIRHSTREKDRG
PTKATTTKLVYQIFDTFFAEQIEKDDREDKENAFKRRRCGVCEVCQQPECGKCKACKDMVKFGGSGRSKQ
ACQERRCPNMAMKEADDDEEVDDNIPEMPSPKKMHQGKKKKQNKNRISWVGEAVKTDGKKSYYKKVCIDA
ETLEVGDCVSVIPDDSSKPLYLARVTALWEDSSNGQMFHAHWFCAGTDTVLGATSDPLELFLVDECEDMQ
LSYIHSKVKVIYKAPSENWAMEGGMDPESLLEGDDGKTYFYQLWYDQDYARFESPPKTQPTEDNKFKFCV
SCARLAEMRQKEIPRVLEQLEDLDSRVLYYSATKNGILYRVGDGVYLPPEAFTFNIKLSSPVKRPRKEPV
DEDLYPEHYRKYSDYIKGSNLDAPEPYRIGRIKEIFCPKKSNGRPNETDIKIRVNKFYRPENTHKSTPAS
YHADINLLYWSDEEAVVDFKAVQGRCTVEYGEDLPECVQVYSMGGPNRFYFLEAYNAKSKSFEDPPNHAR
SPGNKGKGKGKGKGKPKSQACEPSEPEIEIKLPKLRTLDVFSGCGGLSEGFHQAGISDTLWAIEMWDPAA
QAFRLNNPGSTVFTEDCNILLKLVMAGETTNSRGQRLPQKGDVEMLCGGPPCQGFSGMNRFNSRTYSKFK

NSLVVSFLSYCDYYRPRFFLLENVRNFVSFKRSMVLKLTLRCLVRMGYQCTFGVLQAGQYGVAQTRRRAI
ILAAAPGEKLPLFPEPLHVFAPRACQLSVVVDDKKFVSNITRLSSGPFRTITVRDTMSDLPEVRNGASAL
EISYNGEPQSWFQRQLRGAQYQPILRDHICKDMSALVAARMRHIPLAPGSDWRDLPNIEVRLSDGTMARK
LRYTHHDRKNGRSSSGALRGVCSCVEAGKACDPAARQFNTLIPWCLPHTGNRHNHWAGLYGRLEWDGFFS
TTVTNPEPMGKQGRVLHPEQHRVVSVRECARSQGFPDTYRLFGNILDKHRQVGNAVPPPLAKAIGLEIKL
CMLAKARESASAKIKEEEAAKD

>DNA (cytosine-5)-methyltransferase 1 [Rattus norvegicus]
MPARTAPARVPALASPAGSLPDHVRRRLKDLERDGLTEKECVKEKLNLLHEFLQTEIKSQLCDLETKLHK
EELSEEGYLAKVKTLLNKDLCLENGTLSLTQKANGCPANGSRPTWKAEMADSNRSPRSRPKPRGPRRSKS
DSETMIEASSSSVATRRTTRQTTITSHFKGPAKRKPKEDSEKGNANESAAEERDQDKKRRVAGTESRASR
AGESVEKPERVRPGTQLCQEEQGEQEDDRRPRRQTRELASRRKSREDPDREARPGTHLDVDDDDEKDKRS
SRPRSQPRDLATKRRPKEEVEQITPEPPEGKDEDEREEKRRKTTRKKPEPLSIPVQSRVERKASQGKASA
IPKLNPPQCPECGQYLDDPDLKYQQHPVDAVDEPQMLTNEALSVFDSNSSWFETYDSSPMHKFTFFSVYC
SRGHLCPVDTGLIEKNVELYFSGVAKAIHEENPSVEGGVNGKNLGPINQWWISGFDGGEKALIGFSTAFA
EYFLMEPSPEYAPIFGLMQEKIYISKIVVEFLQSNPDAVYEDLINKIETTVPPSAINVNRFTEDSLLRHA
QFVVSQVESYDDAKDDDETPIFLSPCMRSLIHLAGVSLGQRRATRRTVINSAKVKRKGPTKATTTKLVYQ
IFDTFFSEQIEKDDKEDKENTMKRRRCGVCEVCQQPECGKCKACKDMVKFGGTGRSKQACLKRRCPNLAV
KEADEDEEADDDIPELPSPKKLHQGKKKKQNKDRISWLGEPVKIEENRTYYWKVSIDEETLEVGDCVSVI
PDDPSKPLYLARVTALWEDKNGQMFHAHWFCAGTDTVLGATSDPLELFLVGECENMQLSYIHSKVKVIYR
GPSPNWAMEGGMDPEAMLPGAEDGKTYFYQFWYSQDYARFESPPKTQPTEDNKHKFCLSCIRLAELRQKE
MPKVLEQLEEVDGRVYCSSITKNGVVYRLGDSVYLPPEAFTFNIKMASPMKRSKRDPVNENLYPEHYRKY
SDYIKGSNLDAPEPYRIGRIKEIHCGKKKGGKVNEADIKIRLYKFYRPENTHKSIQATYHADINLLYWSD
EEAVVDFSDVQGRCTVEYGEDLLESIQDYSQGGPDRFYFLEAYNSKTKSFEDPPNHARSPGNKGKGKGKG
KGKGKPQVSEPKEPEAAIKLPKLRTLDVFSGCGGLTEGFHQAGISETLWAIEMWEPAAQAFRLNNPGTTV
FTEDCNVLLKLVMAGEVTNSLGQRLPQKGDVEMLCGGPPCQGFSGMNRFNSRTYSKFKNSLVVSFLSYCD
YYRPRFFLLENVRNFVSFRRSMVLKLTLRCLVRMGYQCTFGVLQAGQYGVAQTRRRAIILAAAPGEKLPL
FPEPLHVFAPRACQLSVVVDDKKFVSNITRLSSGPFRTITVRDTMSDLPEIQNGASAPEISYNGEPQSWF
QRQLRGSHYQPILRDHICKDMSALVAARMRHIPLSPGSDWRDLPNIQVRLRDGVITNKLRYTFHDTKNGC
SSTGALRGVCSCAEGKTCDPASRQFNTLIPWCLPHTGNRHNHWAGLYGRLEWDGFFSTTVTNPEPMGKQG
RVLHPEQHRVVSVRECARSQGFPDTYRLFGNILDRHRQVGNAVPPPLAKAIGLEIKLCLLASAQESASAA
VKGKEETTTED

>DNA (cytosine-5)-methyltransferase 1 [Sus scrofa]
MPARTAPARVAALASRAFSLPDDVRRRLKDLERHSLTEKECVKEKLNLLHEFLQTEIKNQLCDLETKLHK
EELSEEGYLAKVKSLLNKDLSLENGAHAFSREVNGYLENGSQTSGEDRRVEMAEENKSPKPVSRLGTPRR
SKSDGEAKSAEVSSSPRITRQTTRQTTITSHFTRGPGKRKPEEDTAKAKPDSPVEEEEKDQEEKRRKVTS
RDSVAGLLPTEEPERVRPGTHMEEDDKEEKRLRSQTKELTPKQKIKEELDRSTRPGGAQPGTNEEDKDEK

RHRSQPKGLAGKRRPEEKEPERIKPQVSDEKDEDEKEEKRRRTTYKEPTEKKLARTKTAVVSTKADPLKC
VQCGQYLDDAELKYEQHPPDAVEEIQLLTNERLSIFDANESGFESYEALLQHKLTGFSVYCKRGHLCPID
TGLIEKDVELFFSGSAKPIYEDDPSLEGGVNGKNLGPINEWWITGFDGGEKALIGFSTSFAEYILMDPNP
EYAPLFSVMQEKIYISKIVVEFLQNNPDSTYEDLINKIETTVPPSVLNLNRFTEDSLLRHAQFVVEQVES
YDQAGDSDEQPIFLTPCMRDLIKLAGVTLGKRRAERRRTIGHSTKEKDKGPTKATTTKLVYQIFDTFFAE
QIEKDDKEDKENAFKRRRCGVCEVCQQPECGKCKACKDMVKFGGSGRSKQACQERRCPNMAMKEADDDEE
VDDNIPEMPSPKKMHQGKKKKQNKDRISWIGEAVKTDGKKIYYKKVCIDSETLEVGDCVSVIPDDSSKPL
YLARVTALWEDSSNGQMFHAHWFCAGIDTVLGATSDPLELFLVDEGEDMQLSYIHSKVKVIYKPPSENWA
LEGGMDPEALMSKDDGKTYFYQLWYDQEYARFESPPKTQPTEDNKFKFCVSCARLAEMRQKEVPRVMEQL
EDLDGRVLYSSATKNRIQYRVGDGVYLPPEAFTFNIKLSSPVKGPRKEPVDEDLYPEHYRKYSDYIKGSN
LDAPDAYRIGRIKEIFCTKKSNGKPNETDIKIRLNKFYRPENTHKSTPASYHADINLLYWSDEEAVVDFK
SVQGRCTVEYGEDLPECLQDFSAGGPDRFYFLEAYNAKSKSFEDPPNHARSPGNKGKGKGKGKSRTKSQT
CEPSELETEIKLPKLRTLDVFSGCGGLSEGFHQAGISETLWAIEMWDPAAHAFRLNNPGSTVFTEDCNVL
LKLVMAGEVTNSRGQKLPQKGDVEMLCGGPPCQGFSGMNRFNSRTYSKFKNSLVVSFLSYCDYYRPRYFL
LENVRNFVSFKRSMVLKLTLRCLVRMGYQCTFGVLQAGQYGVAQTRRRAIILAAAPGEQLPLFPEPLFAP
RACQLSVVVDDKKFVSNITRLSSGPFRTITVRDTMSDLPEIRNGASAQDISYNGEPQSWFQRQLRGSQYQ
PILRDHICKDMSALVAARMRHIPLAPGSDWRDLPNIEVRLSDGTLARKLRYNYHDKKNGCSSTGALRGVC
SCVEVGKACDPAARQFNTLIPWCLPHTGNRHNHWAGLYGRLEWDGFFSTTVTNPEPMGKQGRVLHPEQHR
VVSVRECARSQGFPDTYRLFGNILDKHRQVGNAVPPPLAKAIGLEIKRCMLAKARESASVKVKEEETTKD

>DNA methyltransferase (cytosine-5) 1, isoform CRA_b, partial [Mus musculus]
PLPIGFRAREKAGVSFRAVLSSATCKMPARTAPARVPALASPAGSLPDHVRRRLKDLERDGLTEKECVRE
KLNLLHEFLQTEIKSQLCDLETKLHKEELSEEGYLAKVKSLLNKDLSLENGTHTLTQKANGCPANGSRPT
WRAEMADSNRSPRSRPKPRGPRRSKSDSDTLFETSPSSVATRRTTRQTTITAHFTKGPTKRKPKEESEEG
NSAESAAEERDQDKKRRVVDTESGAAAAVEKLEEVTAGTQLGPEEPCEQEDDNRSLRRHTRELSLRRKSK
EDPDREARPETHLDEDEDGKKDKRSSRPRSQPRDPAAKRRPKEAEPEQVAPETPEDRDEDEREEKRRKTT
RKKLESHTVPVQSRSERKAAQSKSVIPKINSPKCPECGQHLDDPNLKYQQHPEDAVDEPQMLTSEKLSIY
DSTSTWFDTYEDSPMHRFTSFSVYCSRGHLCPVDTGLIEKNVELYFSGCAKAIHDENPSMEGGINGKNLG
PINQWWLSGFDGGEKVLIGFSTAFAEYILMEPSKEYEPIFGLMQEKIYISKIVVEFLQNNPDAVYEDLIN
KIETTVPPSTINVNRFTEDSLLRHAQFVVSQVESYDEAKDDDETPIFLSPCMRALIHLAGVSLGQRRATR
RVMGATKEKDKAPTKATTTKLVYQIFDTFFSEQIEKYDKEDKENAMKRRRCGVCEVCQQPECGKCKACKD
MVKFGGTGRSKQACLKRRCPNLAVKEADDDEEADDDVSEMPSPKKLHQGKKKKQNKDRISWLGQPMKIEE
NRTYYQKVSIDEEMLEVGDCVSVIPDDSSKPLYLARVTALWEDKNGQMMFHAHWFCAGTDTVLGATSDPL
ELFLVGECENMQLSYIHSKVKVIYKAPSENWAMEGGTDPETTLPGAEDGKTYFFQLWYNQEYARFESPPK
TQPTEDNKHKFCLSCIRLAELRQKEMPKVLEQIEEVDGRVYCSSITKNGVVYRLGDSVYLPPEAFTFNIK
VASPVKRPKKDPVNETLYPEHYRKYSDYIKGSNLDAPEPYRIGRIKEIHCGKKKGKVNEADIKLRLYKFY
RPENTHRSYNGSYHTDINMLYWSDEEAVVNFSDVQGRCTVEYGEDLLESIQDYSQGGPDRFYFLEAYNSK
TKNFEDPPNHARSPGNKGKGKGKGKGKGKHQVSEPKEPEAAIKLPKLRTLDVFSGCGGLSEGFHQAGISE

TLWAIEMWDPAAQAFRLNNPGTTVFTEDCNVLLKLVMAGEVTNSLGQRLPQKGDVEMLCGGPPCQGFSGM
NRFNSRTYSKFKNSLVVSFLSYCDYYRPRFFLLENVRNFVSYRRSMVLKLTLRCLVRMGYQCTFGVLQAG
QYGVAQTRRRAIILAAAPGEKLPLFPEPLHVFAPRACQLSVVVDDKKFVSNITRLSSGPFRTITVRDTMS
DLPEIQNGASNSEIPYNGEPLSWFQRQLRGSHYQPILRDHICKDMSPLVAARMRHIPLFPGSDWRDLPNI
QVRLGDGVIAHKLQYTFHDVKNGYSSTGALRGVCSCAEGKACDPESRQFSTLIPWCLPHTGNRHNHWAGL
YGRLEWDGFFSTTVTNPEPMGKQGRVLHPEQHRVVSVRECARSQGFPDSYRFFGNILDRHRQVGNAVPPP
LAKAIGLEIKLCLLSSARESASAAVKAKEEAATKD

>DNA methyltransferase 1 L homeolog [Xenopus laevis]
MPAQSTSLALPADVRKRLKDLKRDQDGMTEKEHVQQKLSLVLGFLEADARNKLNDLESKLSSEELSEEGY
LTKVKSLLGKQLSFENVDLALNGETNGCSTNGTCGSDEEDVQLSESNTSGVKNRKPRKSKVNGENKKSPA
RARPSRSTAGKQPTILSMFSKGSTKRKSSDDEKDTDVPADADQPEEKEKEEKRIKIEVNESEDKRSDAEE
GKKAKPVQPPKTPPPKCMDCRQYLDDPDLKYFQGDPDDALDEPEMLTDERLSLFESNEDGFESYDDLPQH
KVTCFSVYDKRGHLCPFDSGLIEKNVELYFSAVVKPIYDDSPSLDGGVRAKKLGPINAWWITGFDGGEKA
LIGFTTAFADYILMDPSEEYSSIFALIEEKIYMSKIVVEFLQNNPDVSYEDLLNKIETAVPPSALNFNRF
TEDSLLRHAQFVVEQVESYDEAGDSDEQPVIVTPCMRDLIKLAGVTLGKRRAARRQTIRHPTKIEKDKGP
TKATTTRLVYQIFDTFFFEQIEKDAEKENGIKRRACGVCEVCQQPDCGQCKACQAMLKFGGAGRTKQACM
QRRCPNLAVKEADEDEEVEDVLPEMPSPKKILQGKKKKLEKKNRISWVGDPVKTEGKKEYYLKVSIDSEI
LEVGDCVSVSPDNPTEPLYLARITSMWEEGCGQMFHAHWFCLGTDTVLGATSDPLELFLVDECEDMQLSY
IHGKVNVLYKAPSDNWFMEGGTDTEIKVVEDDGNTYFYQLWYDPEYARFETPPKPQSTEDNKYKFCTSCA
RLDEIRQKEIPRVSNPVEELDSKICYSTATKNDVHYKVGDGVLHFPDAFSFSVKLGSPMKRPQRKDDVDE
DLYPEYYRKSSDYIKGSNLDAPEPYRLGRIKEIFCNKRSNGKPNEADIKLRIYKFYRPENTHKGMKASYH
SDINMVYWSDEEAVVEFKAVQGHCTVEYGEDLTESIQEYSAGGSDRFYFLEAYNAKTKSFEDPPNHARGA
VNKGKGKGKGKGKGKTPSKSENEQLNSGDKLPKLRTLDVFSGCGGLSEGFHQAGISETNWAIEMWEPAAQ
AFRLNNPGTTVFTEDCNILLKLVMSGEKTNSLGQRLPQKGDVEMLCGGPPCQGFSGMNRFNSRTYSKFKN
SLVVSYLSYCDYYRPKYFLLENVRNFVSFKKSMVLKLTLRCLVRMGYQCTFGVLQAGQYGVAQTRRRAIV
LAAAPGEKLPMFPEPLHVFAPRACTLSVVVDEKKYVSNTTRTNSSLFRTITVRDTMSDLPEIRNGASALE
ISYNGEPQSWFQRQIRGSQYQPILRDHVCKDMSALVAARMRHIPLAPGSDWRDLPNMEVRLSDGTTSRKL
RYTHHDKKNGRSGTGALRGVCCCSEGKQCDPADRQFGTLIPWCLPHTGNRHNHWAGLYGRLEWDSFFSTT
VTNPEPMGKQGRVLHPEQHRVVSVRECARSQGFPDTYRLFGNILDKHRQVGNAVPPPLSRAIGLEIKSCV
LTRMKENGTETVKAEKMETD

>DNA cytosine-5 methyltransferase [Bombyx mori]
MPTSTITCNSITQVCMDKILDGDEIIKENSKRKRSCEEIVSANNKRSKTEDNDGHQISQDSTSNDSIENL
NIPAKNTSLIVSENINNVYKNYDEVENKPVYKNGDPIVVEIDNKDKPGSVNNNHDMIDDSETISNIKNSN
NIIPDTEKCNICGQFLNNSDLIYYQGHPQDAVEEYIALTNDKLVLSSGEDGDIMERPQTNITGFTIFDEQ
GHLCPIDGGLVENDVRIYMSGYLKSICSDSSEIDEESIPVKDVGPIIEWFIHGFDGGDRNCITLSTEFGE
YNLLKPSEAYTPLMNNLYEKIWLSKVVVEFLEEYHYLQPSYEDLLEVVRDFSIPELNNKKMTEEMLHKHA

QFVCDQVVSLEIEEDDEPLITLPCMRELIKLMGIKFGKRKIRTQIQYKKTDKKAWTKATTTPLVRKTFES
FFSNQLDKTNHELVLRRKRCGVCEACQLPDCGECNACRAMAKFGGHGRTKKACVRRLCPNMAVEQAEDSD
PDDEDEYQQISEKKQDKIDDAVPVKLTGSNSKNLKWIGEPVKADATKIYYEKVEIDGAELCNGDFVMIET
SQTNIPTLVAKVVYMWKEIHNPKSGYFHGEVFIRASDTVLGEVSDPREVFLGDRCCHGAPLSSILRKANI
ERKETSADWFKLGGKEVDDEHFEDDGRTYFYSKYYDRFTSRFEDLPPDPACPNALRKHRFCPSCERKTKR
DARNIPKVFEKLIVKSEIVSEQNRSEWSYVKWQDFDYKKGCGVFLKPGTFKLKNSMTKANTVAKPRFEKV
DETIYPEYYRKNDSNTRGSNIDTGEPFCVGYIAAVTAASEGPLVVPQDIYLKVNVLLRPENTSSKFPQHE
DTNVLYWTTEIREIPFSTVVGHCHLIYEQNVPQNISLQEWLGNDPCRFYFRMAYCKSTGEFTDLPQNAIS
VGRTDRTKDKGKGKGKSTKTIETVPAKVVEEKIRPLRTLDVFAGCGGLSEGLHQAGVAECKWAIENVEAA
SHAYSLNNKSCIVFNEDCNALLKTVMSGAKHSANGLRLPMQGEVELLCGGPPCQGFSGMNRFNSREYSNF
KNSLVASYLSFCDYYRPKYFILENVRNFVAFKKGMVLKLTLRALLDMGYQCTFGILQAGNYGVPQTRRRL
IILAAAPGYNLPFYPEPTHVFSRRACTLTTTIDGKRFSTNIHWDESAPKRTCTIQDAMSDLPQICNGANK
IEIEYGSMPESHFQRLVRSNDENSKLRDHICKNMAPLIQARISRIPTTPGSDWRDLPNISVTLSDGTKCK
VLQYRYDDKRNGRSSSGAVRGVCACASGRACSPLDKQENTLIPWCLPHTGNRHNHWAGLYGRLSWGGYFS
TTVTDPEPMGKQGRVLHPDQHRVVSVRECARSQGFPDTYLFAGSVQDKHRQIGNAVPPPLGAALGREIKK
ALTLSLTTS

>DNA methyltransferase-2 [Arabidopsis thaliana]
MAEQELQRINEKKPWQVLEFYSGIGGMRYSLMASGIVSEVVEAFEINDSANDVYQHNFKHRPYQGNIQSL
TAADLDKYNADAWLLSPPCQPYTRQGLQKHSGDARAFSFLRILELIPHTTKPPQMLFVENVVGFETSDTH
MEMIGTLTKLDYVTQEFILSPLQFGVPYSRPRYFCLAKRKPLPFKSQHSNNKLLWSPDPLYGRDDQVEFG
KCQAEEGLDKLLEFCKPVEKFLELAAHVDGEPSSVDDSENGSKDCCGQEGDSVPDSVHQYLVPVSLIERW
GNAMDIVYPDSKRCCCFTKSYYRYVKGTGSLLATVQPKIKGKESCLKEQRLRYFTPREVANFHSFPEDFE
FPKHISLRQRYAMLGNSLSVAVVAPLLRYLFDS

>DNA methyltransferase-2 [Spodoptera frugiperda]
MSHKILELYSGIGGMHCAWKESGLDGEIVAAVDINTVANSVYKHNFPETNLLNRNIQQLTPQVIKKWNVD
TILMSPPCQPFTRNGKYLDDNDPRTNSFLYLIGILDQLDNVDYILMENVKGFENSTVRNLFIDKLKECNF
IYQEFLLCPSTVGVPNSRLRYYCTARRNNLTWPFKRRDEIITRLPKDFGVPHSLESIIEEDVDEKFLVPE
KMLRCAKVFDICYKTSKRSCCFTKAYTHYADGTGSIFTDKPREVVQKCYEEAKQNEIGGEKFVELFKELK
LRYFTPKEVLMIMCFPKSYNLPTNISMKQCYRLLGNSVNVKVISELLKILFE

>(cytosine(38)-C(5))-methyltransferase [Sus scrofa]
MEPLRVLELYSGIGGMHQALRESCIPAQVVAAIDVNTVANEVYKYNFPHTQLLAKTIEGVTLEEFDRLSF
NMVLMSPPCQPFTRIGLQGDVTDPRTNSFLYILDILPRLQKLPKYILLENVKGFEVSSTRDLLIQTIENC
GFQYQEFLLSPTSLGIPNSRLRYFLIAKLQSEPFPFQAPGQVLMEFPQMESEHPQKHAIDAQSKIEEKKI
ERNICLDSSAQCSGKEAILFKLETAGEIDRKHQQDSDLSVQMLKGFLEDDIDMNSYFLPPKSLLRYALLL
DIVKPTSRRSMCFTKGYGRYIEGTGSVLQTSEDVQIENIYKSLTSLSPEEKIMKLLMLKLRFFTPKEIAN

LLGFPPEFGFPEKITVKQRYRLLGNSLNVHVVAKLIKILYASVLSSSARWFQYSIISK

>methyltransferase 2, isoform C [Drosophila melanogaster]
MVFRVLELFSGIGGMHYAFNYAQLDGQIVAALDVNTVANAVYAHNYGSNLVKTRNIQSLSVKEVTKLQAN
MLLMSPPCQPHTRQGLQRDTEDKRSDALTHLCGLIPECQELEYILMENVKGFESSQARNQFIESLERSGF
HWREFILTPTQFNVPNTRYRYYCIARKGADFPFAGGKIWEEMPGAIAQNQGLSQIAEIVEENVSPDFLVP
DDVLTKRVLVMDIIHPAQSRSMCFTKGYTHYTEGTGSAYTPLSEDESHRIFELVKEIDTSNQDASKSEKI
LQQRLDLLHQVRLRYFTPREVARLMSFPENFEFPPETTNRQKYRLLGNSINVKVVGELIKLLTIK

>DNA (cytosine-5-)-methyltransferase 3 [Danio rerio]
MVADVKIGDDKQSLCELLDWLNGLLQATFSQVEDTCSGAAFCQLMDIIQPGSIDVTKVNFTAEENLDILN
NYNLLQEAFSKAQIQKELELTLLVNGDIMTTCDLLTWFKDMYDHNFAKQKCNPQVAFIKPEVVSLKSSRE
FETIEKENVSSLYNTEETSSNQKTQHVEKTSQESVSWSPLTSFIRKYGSSTLTDDESNNVNSKDCPGQKS
FGDITPFWRQTPYCLYLLHGVELEDDKKASVLLLGFFDKETGENKIRLLDVVYPTKESTEDICNYILDTL
RKIGIPLFNMAILYSDFPDHEHLVAGLQLMKAEIVSLCGLTDLTGQVCHSGVEKIEFSDLILNLITEIYK
HFPSFPADLQALLEDVEGSDIDNLTSQCSLFWRIIKKIPLAWSHLEKYFGSLGTEEEAVCLLLEDPKIKL
NVLFLTHALQPLCDFQEIIDQGASVLQLLQDASKLLRLYTQSFLRPKAAEYFHRRGKTSLVQETVGHLPK
GEVAVGEQAADFLQQHSEELFDYLETFHSSIISFYTTVTVNIVKRLPLPDSTLRNLSLVLSPGKKLEVTG
KMVQDLGVGFGVCIRPDNVSLLTDEFLEYQLIEGGDTGSVDQPTEKYWQTELKIMGNASNFGKLIVSLLA
LPKTLKKEIIFKQMFQQTDYLKMMRKEDCEEKDMMEDDVTDSSSYKSAPSHLSPETQGSSISDVIDLTEM
DEIGPVEIEDIAPMDVDDIVSISSDSETENQKVNVPHVSIVLDDDDDDDDEMTDDDDDYGCEAGEVMWKY
SKNKGNTQNEMTDNTYQGGFSVGEMVLGPIEGFGLWPGLVQSWDSERPCGSMRKVIFFGNGMQTEVQADS
LLPFSSLAKCFCSNSFATVMAYKDAIFSSLQVASRRSRMFFSPESESKDELLRVMLNWAFGGFEPLGADG
LQPQAEYSVKVKKGKRKNPTGKLFNLTVPLNKIPESLDLNNGLVDLGTTDADKKRLYSKWNGRSMQTVKI
RRKYKQRNKNIIPTVQIESRQNSQKRHQMVHEFLKNKRKIEDFCLSCGSMSVDIIHPLFEGKLCTNCKFN
FTETLYRYDEDGYQSYCTVCCSGMEVILCGHDSCCRSFCVDCLDILVCQGTFDQLKNVDPWTCYLCAPET
SSGALKPRHDWSIRVQEFFANDTGMEFEPHRVYPSIPAIQRRPIRVLSLFDGIATGYLVLRDLGFKVEKY
VASEIDEESITISMVNHDGKITQVDDVKNITKKHIEQWGPFDLLIGGSPCNDLSIVNPARKGLYEGTGRL
FFEYYRLLNVLKPKEDDPQPFFWLFENVTFMQTHVKADICRFLECNPVLVDAVKVSPAHRARYFWGNIPG
MNRPIIASQNDKLCLQECLEPGRTAKYEKVRTITTRQNSLKQGTNDAHFPVTMNGKDDHIWITELEKIFG
FPKHYTDVKSMGRPQRQRVLGKSWSVPVIRHLLAPLKDYFACDEFPVK

>DNA methyltransferase 3 [Apis mellifera]
MLSEEGKLWVYWIGEARISLLNEKTQIEPFSCNLKARLTQNLNVPRIRAIDATMQMLRKKLGGTLTKPYF
TWIESNFPKNMIEMLDEIKFYPYPVKMQQRLDHLREKNAKVTERYLLDQKRENQEKKLAEKSKDSPQKVN
VDLTLLPLKEQKPGIIAWAKIAGHNWWPAMIIDYRDCCMREPTFGCQWIMWYGDYKLSEVHHQLFLRFDK
GMEKMRDYTSNTKKHIYLVGVLQASKDYCSRLGFDTSNWTLDDAFEYFSKPNHYDYASSANTWRREDSVK
IYDKYSARIAEKLNELKDNPNVDDQRANDINNSDDLRSAIKGEISFDSLCLKCLRVSNDEMDIHPFFEGS

LCKDCSERYKPCMFVFGNDSKCFYCTVCAASGMVIICDKEDCPRVYCTACMKHLLCPTTYEQVLQEDPWE
CFLCKSRSFTTDTIVRPRANWKDKIINMFRTSCDSNVEHLVAKHNSEKRKIRVLSLFDGLGTGLLVLLKL
GFIVDAYYASEIDQDALMVTASHFGDRILQLGNVKDITCNTIKEIAPIDLLIGGSPCNDLSLANPARLGL
HDPRGTGVLFFEYRRILKLVRKLNNERHLFWLYENVASMPSEYRLEINKHLGQEPDVIDSADFSPQHRLR
LYWHNFPIEPRLLSSQREQDVQDILTPHCQRYSLVKKIRTVTTKVNSLKQGDGKLALKPILMKDESDSLW
ITELEEIFGFPRHYTDVKNLSATKRQRLIGKSWSVQTLTAIFESLCPFFERDIVEIEG

>DNMT3 [Daphnia pulex]
MNSDSDDCYDSDATYFCGSDDDDGDGNFGVEADGLLLEKWIPPLFYLAKGHLQTIHNDVNKLENVCLACW
EKGTCPHPFFVGALCQQCKQRLLRTMFSKTVDGFHLYCTICGSKSNACVSCSNGKCYRKYCVNCLNIWTD
HSDKLIKNSDKWFCFLCLPEPNQILQAHHNWAQKVLLFHEPLAVYASRALYWKRQPLRVLSLFDGIGTGL
VALRKLGIEVEVYYASEVLTAAATVSRTRLGGVLHHIGSVGEVTQQRLEEISPIHLLIGGSPCNDFSAIN
RFPKDFYDPRGYSRYFFDFVRVLNLMRKINGQHQHLLWLFENVASMPQHYRETISRHLDCQPAVIDAKNF
SPQLRRRLFWGNIPGLFTVHEQQLTQDGESLSLEKSLMPNSGRRAAQEKIRTLTTNTNSLLQGRTENCDE
DDNLQTDVLWLQEIEHVFGLPRHFTDVGNMSRTDRQKLLGHAWSVPVIASIFSNLKAYTV

>DNA (cytosine-5)-methyltransferase 3B [Danio rerio]
MRKEEIKKSTEIVMPSNKYPSAESDKMTATAAMNRDTSVGDGLSENDSGLEMTSENSPLTPAEPPSPFCP
KQNGGAASPADESVNSIRRKRSRKRSDTEEDSAWDSSYSEEKAEVGSGCETGLRQRPRPRTIFQAGLTAH
SKPRSRERGHSKEDHSDLVASVPEGPALELMEQDSKDSAQSSTTSTSTTETASQPEYKDNKGFGIGELVW
GKIKGFSWWPGMVVTWRATGRRQASHGMRWLQWFGDGKFSEVSADKLDSITAFPKFFNQSSYTKLASYRR
AIFQALEVASLRAEKTFPPSEADSLEEQVKPMLDWAHGGFLPKGQEGLKPKENAEYCVFPLASESSTLLE
SSPPEFPPSAKRARLPLNKAKPGIEEVYSREQMVNEVLKNHRSIEEFCLSCGKTRVATFHPLFEGGLCLT
CKDAYLENSYMYDDDGYQSYCTVCCGGREMLLCGNANCCRCICVDCLDILVGAGAANSARNLDPWRCYMC
QPLQQYGVLKKRHDWSLKLQEFFVNDSGQEFESPKIYPAVPAEQRRPIRVLSLFDGIATGYLVLRDLGFK
VDLYIASEVCEDSISVGAVRHEGKIQYVHDVRNITRKNIAEWGPFDMVIGGSPCNDLSIVNPARKGLYEG
TGRLFFEFYRLLSEAKPKEGEDRPFFWMFENVVAMSVNDKRDISRFLECNPVMIDAIEVSAAHRARYFWG
NLPGMKRPLCASGMDKLELQDCLEHGRVAKFGKVRTITTRSNSIKQGKDQHFPVMMNGKEDILWCTELER
IFGFPVHYTDVSNMGRGARQKLLGRSWSVPVIRHLFAPLKDYFACE

>chromomethylase 3 [Arabidopsis thaliana]
MAPKRKRPATKDDTTKSIPKPKKRAPKRAKTVKEEPVTVVEEGEKHVARFLDEPIPESEAKSTWPDRYKP
IEVQPPKASSRKKTKDDEKVEIIRARCHYRRAIVDERQIYELNDDAYVQSGEGKDPFICKIIEMFEGANG
KLYFTARWFYRPSDTVMKEFEILIKKKRVFFSEIQDTNELGLLEKKLNILMIPLNENTKETIPATENCDF
FCDMNYFLPYDTFEAIQQETMMAISESSTISSDTDIREGAAAISEIGECSQETEGHKKATLLDLYSGCGA
MSTGLCMGAQLSGLNLVTKWAVDMNAHACKSLQHNHPETNVRNMTAEDFLFLLKEWEKLCIHFSLRNSPN
SEEYANLHGLNNVEDNEDVSEESENEDDGEVFTVDKIVGISFGVPKKLLKRGLYLKVRWLNYDDSHDTWE
PIEGLSNCRGKIEEFVKLGYKSGILPLPGGVDVVCGGPPCQGISGHNRFRNLLDPLEDQKNKQLLVYMNI

VEYLKPKFVLMENVVDMLKMAKGYLARFAVGRLLQMNYQVRNGMMAAGAYGLAQFRLRFFLWGALPSEII
PQFPLPTHDLVHRGNIVKEFQGNIVAYDEGHTVKLADKLLLKDVISDLPAVANSEKRDEITYDKDPTTPF
QKFIRLRKDEASGSQSKSKSKKHVLYDHHPLNLNINDYERVCQVPKRKGANFRDFPGVIVGPGNVVKLEE
GKERVKLESGKTLVPDYALTYVDGKSCKPFGRLWWDEIVPTVVTRAEPHNQVIIHPEQNRVLSIRENARL
QGFPDDYKLFGPPKQKYIQVGNAVAVPVAKALGYALGTAFQGLAVGKDPLLTLPEGFAFMKPTLPSELA

>DNA (cytosine-5)-methyltransferase CMT3 [Malus domestica]
MNXVVNHSYDQFLKPFLSILMAKTIQLLKVFIISPDDHNRXCEQDLEAAMQQVADKTNSIGLALLPVQIH
SXHLPVIFYFLGLSVLLAFTCILFHRLLRPFSPSPLSLCAVRRMACKRKAKAGTSSGSPPESKKLRXTEE
ARVEEEVATTVDKAASPPRNPVTQEVAAADDDGEEARFLGEPMEDEEARKQYPKRYVGKKPKMNGQNNSN
DDEDIIQARCHYTKALVDGINYDLYDDAHSGEKEEPYICKIVEMFEAIGGLLYFTAQWYYRSRDTVIKHC
TTVACGRVFFSDVRDDNPLNCLVEKLHIVRLTLNVDEDLKSKSIPVCNYYCDTKYLLPYSTFVNLXAENM
QTGSDDSTISVEDDVCLDSEVDSKLPNGERAKSEVRLLDLYSGCGAMSTGLCLGAHLANVNLVTRWAVDY
NKYACKSLAQNHPETEVRNEAAEDFLTMLKAWRKLCMCLKLVENDNLEEDVDKSILDFFGKEDDEEEED
EEEEDVSGNVKNDSEVFEVDCVIGVCFGDPKKTEKKGIYFKIHWKGYGPXEDTWEPMNELEHCKQAIKKF
ITEGYRLKKLPLPGDVDVVCGGPPCQGVSGFNRFRNTESPLADDKNQQLEVYMDIVRYLXPKFVLMENVV
DILKFADGFLGRYALGRLVDMNYQVRMGMMAAGAYGLPQFRMRVFLWGARPTEILPQYPLPTHDVVSRGV
TPTQFEGNAVAYDEGHQTQLGKSLFLDDAISDLPAVANNEERDEMPYGVAPKTEFQRLIRLSKEYLMGTS
KDNSIQNVLYDHRPLQLNPDDYARVCEVPKRKGACFRDLPGVRVRGDNKVEWDPEVPRVYLASGKPLIPD
YAMSFVNGSSSKPFARLWWDETVPTVVTRAEPHNQAIMHPEQDRVLTIRENARLQGFXDFYKLSGPIKER
YMQVGNAVAVPVARALGYALGLALKGSAGADPLFXLPANFPNLQDQCTVHTSFHQSNYIKAFTLCTLHYL
LMASASRSSXWLFSLSENWLPMATIIFACGFVGYAVYDAVMATVSELLQRLLVISPLLIVIVVHWLSTGS
QVNISIPGSEPGAIHRAGGSPWGVAFVLFLLFFLISYQPSLHGLLF

>FGSG_10766
MPHIDLDEDDGIDIPPEVLANPNIDWMTSSNQNHGFVVDDVFEDAFAPQESYTNTDHGTPKMWTKLGEMEDEEDDRMSVM
EQVDLSSMSPAPSPPNYDSGPGDLFEGIDVEMLDDIEPEMTIEEDFGLAVDQSGATAELLEQEDGTAVKVDTPDLPLSNS
VRVEVPDALFVIPQSFYEPFEPGVPIDREYKVVAQLLEAAIQEGQDADDFIEFELNDFAIYSKRPRYEHEMCSLHHLDTK
SGHSNLYFDGKLNVGDAAFFVRHVPIRALPIGDYGTLSKHTLRDQIWVQSAMNLKKNIYYRLNRPAKEYRRFFDPFLWVA
DLAKHFVDYLKVMGENKQDVTIFHFRSTFSNWLAKTHNTDPVFASWREQHPSADFRTSIVANLAFLHKEAIGVLDYAGTY
HHTLWAETWEFRQYERLTKTKESDKEARSKPPRSASRTPSSADQVDCDQGKNATDVPQTIVTQYIYDCFNHLPFGDILEA
MSLSDETELLRNELIQKRHLELPAPLHQNAKNVSTAGQQQIKDIQPGDTISTQRDGADSGTKWRRDLSHGFNDVDRWLAL
VQRVRVSKKGVRAFDVIWYYRPVDTLCALMKYPWNNELFLSDHCSCDEPSKISEDEIMGVHEVEFEGTSNTEAEFFCRQT
YVVTERKWITLEKTHLHCLHQRKTTQAPLYQPGETRLIMLDKASGRSEPCEFITFYDEDGTGIYRFRRLLRRHQVDPQTP
NAQPNELVYSENLIEVKNRRILEPCYVRFFKAQETIPTPYDRDGVGNFFYITHEEVIDAETTAKSYLPLEALPSSLRQGH
NPSEPLQKLRGLDLFCGGGNFGRGLEDGGGIEMRWANDYDGKALHTYMANTSGPDAVHPFLGSIDDMQRFAIQGKFAENV
PPVGDVDFISGGSPCPGFSLLTNDKTTVAQRKNQSLVAAFGSFIDLYRPRYGLLENVPGIIQSKATRDQDVFSQLICAIV
GLGYQTQFSFLDASSCGSPQRRSRVFVVFAAPGHHLPQRPHITHAHPEHTRNYGLGSTPTGDPMAERIMPIATPFDFVSA

RAATAGLPPVYDSKPDICVSYPDHRVSIGMTSNLRNKISLIPTRPWGMNFAQAWFGLDCKQAGSGVLSPADRLVFPEEGK
TSLTRGGATSNAYGRQRPDRLIETIVTSQSPSDAKNGRTIHWSENRVLTIMEARRAQGFRDHEVLLGNPADQWKIVGNSV
AREVAVSLGAVFREAWAQSLADEHHAESDVGVATTARGSSKAPVREGVEVSPSMSVDKPTFESASSSNKTPRSRKSRSQS
RKSQTTPTPGPDRSSSSKRSSIAIEVRVSKSPKSEKDDSPSRGTSVASSRTRPSQLRHSVSNE

>FGSG_08648
MVVTHIDIEQREEMEEVIFLTERPTGFPERRRCLSSCTLDGVDTVEVLDAVQVVDAVEVVDYDEIIDLTADLTGLPLSRQ
GELHLEKIQILNTTVQKDSFIRVRKFLFGKYHVKFVLVKTVIRCLSTNTIKIRGIPFIKASEAYSKPHGMMPNEVCMIIY
QDEAKHQEFVDINLSDILQLHHLGSRKRQVEEAFIRIHSSEADTQSKVSDEVLRKRWRGKTNKGGSWIPSNVSNPIDLES
DAEDNKNRLDGQRYTMFDSCSGAGGVSRGALMAGFKIQYAIDKAPEVWETYETNFPDTELFRMPLDEFIAEPNVGHKRVD
ILHFSPPCQFFSPAHTHASVHDDDNIAALFGCNELLQKLRPRVVTVEQTFGLTHDRHGDYFNGLLHDFTQWNYSFRWKVV
KLCTWGAAQDRKRLIIVAAAPGERLPPFPKATHGDEPGLLPYNTIGKALRGIQLEDDLHDPDKVHHFNPPRAPYDPERLA
GTITTRGGDLYYPDGSRKLTLREFASLQGFPRWHLFLGNITSIKRQIGNAFPPVTVRVLYKHIEQWLLKEDGMTPCDDRN
IIAIEEDSEDESPLSPDMMEVDIDERSNHDDCVAEAMVIELT

Figure. S2

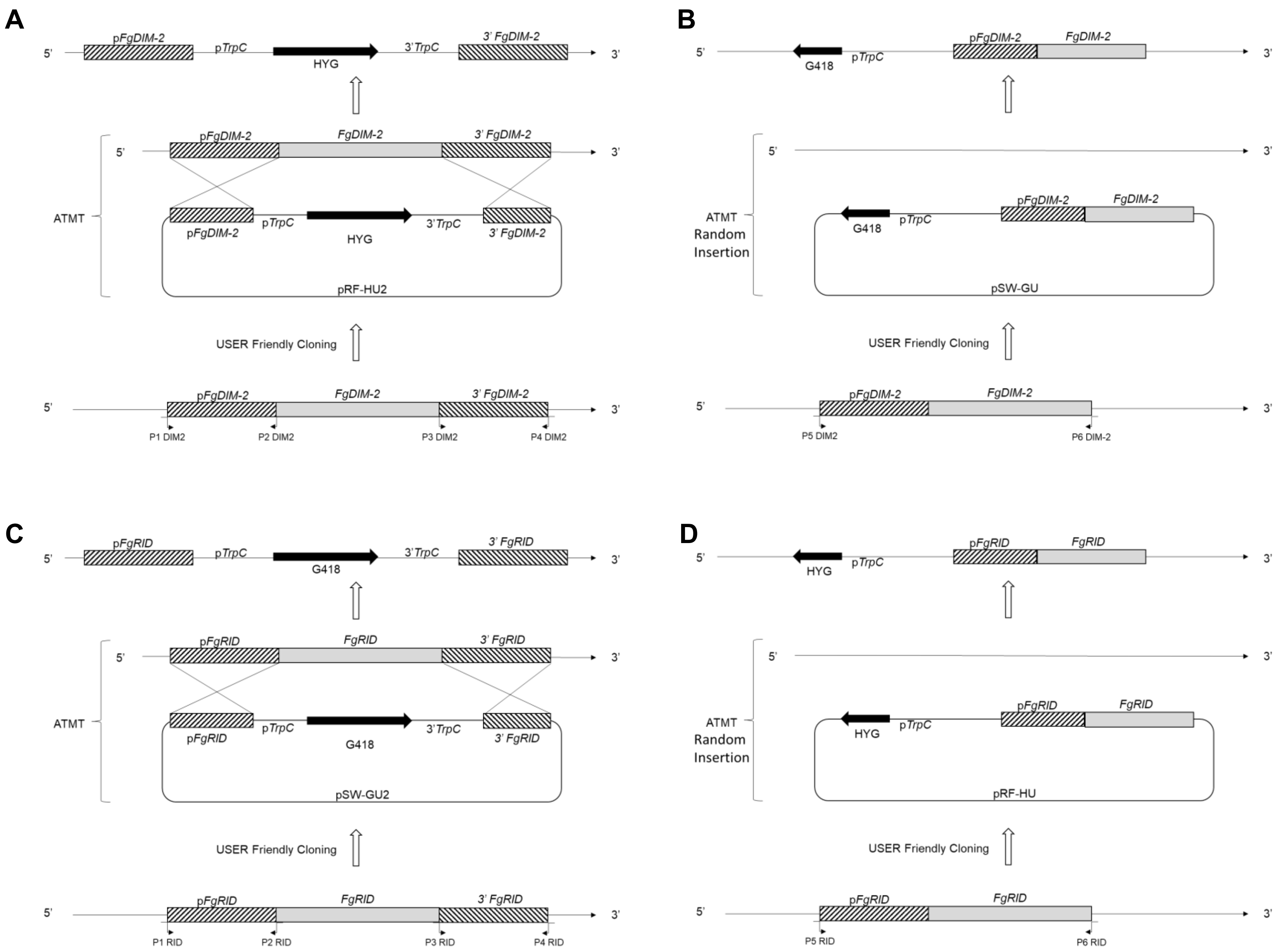
A
pFgDIM-2
pTrpC
HYG
3'TrpC
3' FgDIM-2
ATMT
FgDIM-2
pRF-HU2
USER Friendly Cloning
P1 DIM2
P2 DIM2
P3 DIM2
P4 DIM2
B
G418
ATMT Random Insertion
pSW-GU
P5 DIM2
P6 DIM-2
C
pFgRID
FgRID
3' FgRID
pSW-GU2
P1 RID
P2 RID
P3 RID
P4 RID
D
pRF-HU
P5 RID
P6 RID

Figure S3

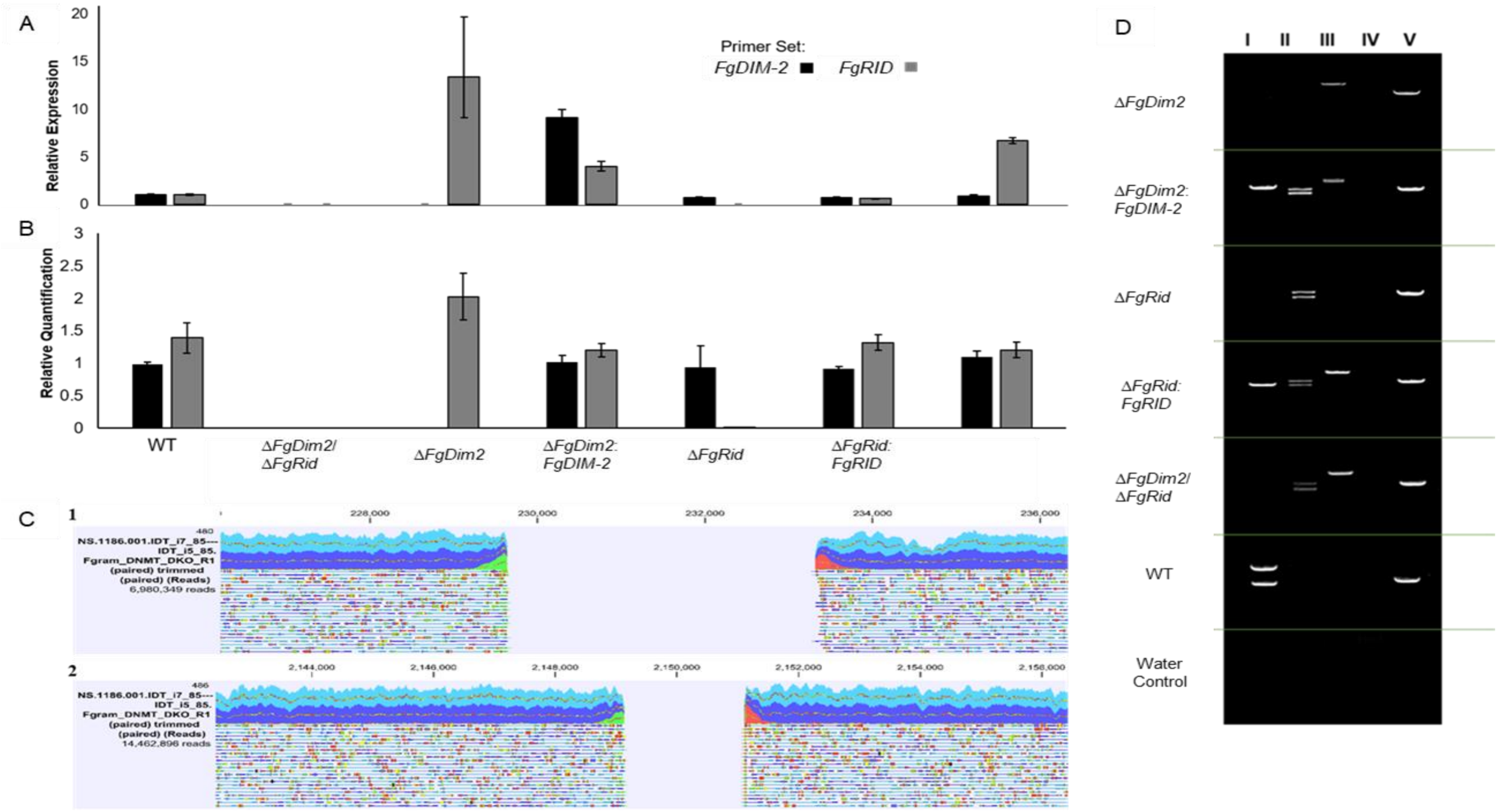
A
B
C
D
Relative Expression
Relative Quantification
Primer Set:
FgDIM-2
FgRID
WT
ΔFgDim2/ ΔFgRid
ΔFgDim2
ΔFgDim2: FgDIM-2
ΔFgRid
ΔFgRid: FgRID
I
II
III
IV
V
Water Control

Figure S4

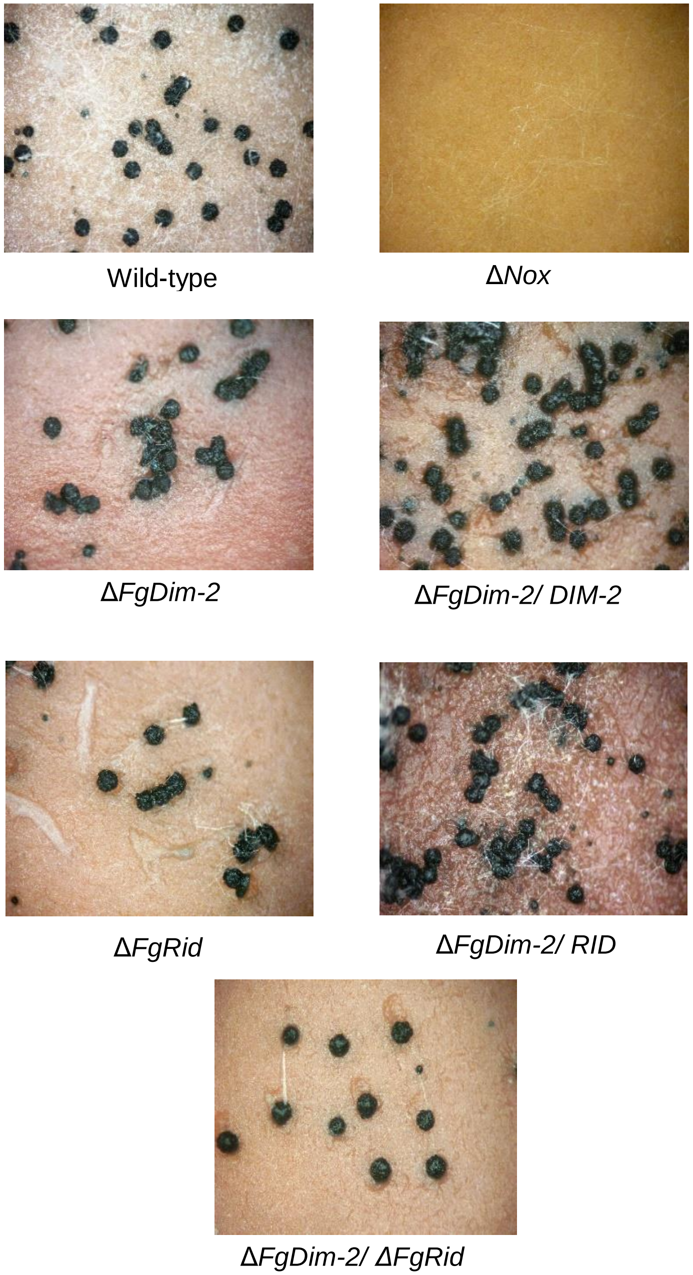
Wild-type
ΔNox
ΔFgDim-2
ΔFgDim-2/ DIM-2
ΔFgRid
ΔFgDim-2/ RID
ΔFgDim-2/ ΔFgRid

Figure. S5

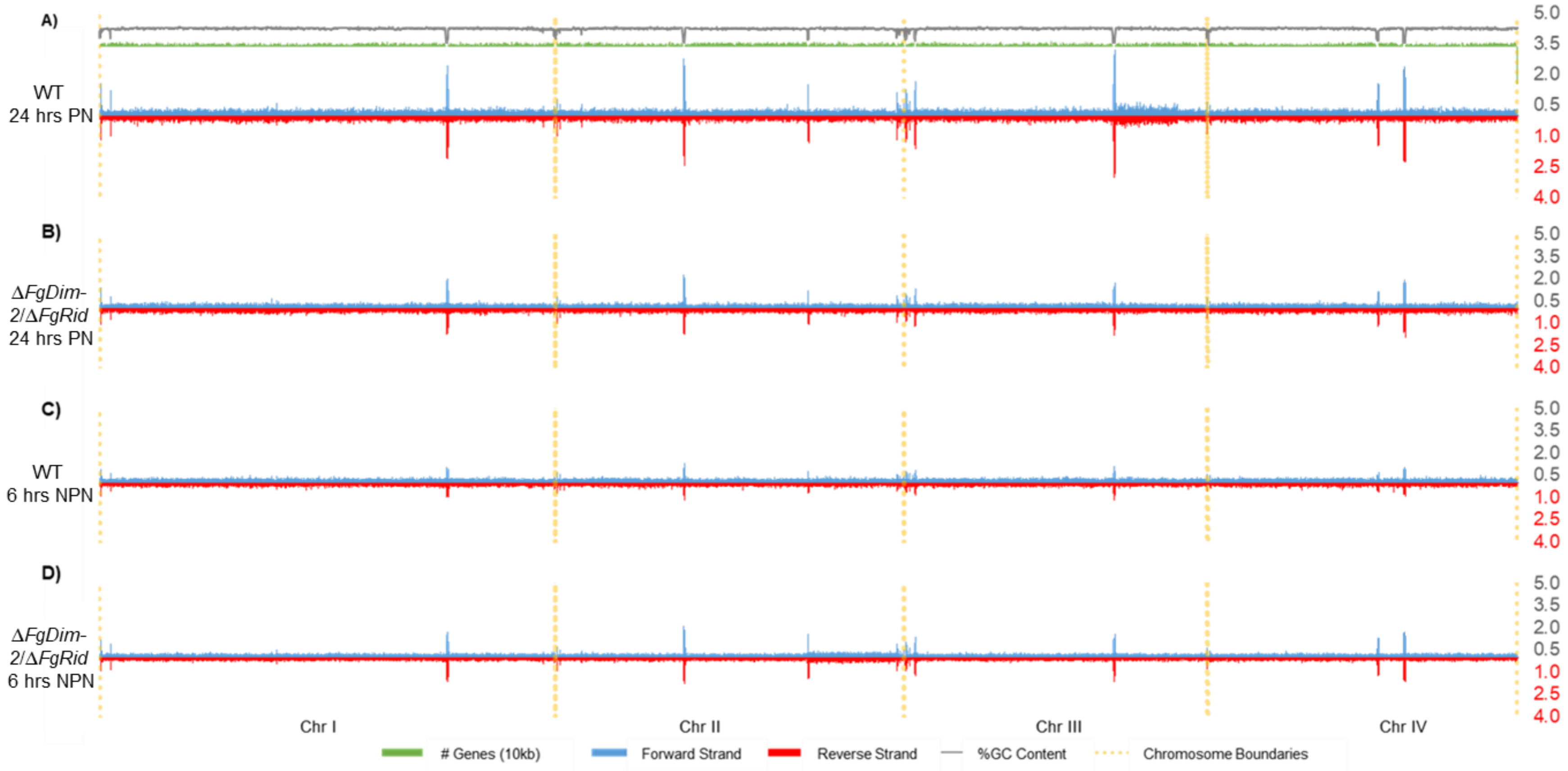
A)
WT
24 hrs PN
B)
ΔFgDim-2/ΔFgRid
24 hrs PN
C)
WT
6 hrs NPN
D)
ΔFgDim-2/ΔFgRid
6 hrs NPN
5.0
3.5
2.0
0.5
1.0
2.5
4.0
Chr I
Chr II
Chr III
Chr IV
Genes (10kb)
Forward Strand
Reverse Strand
%GC Content
Chromosome Boundaries

Figure S6

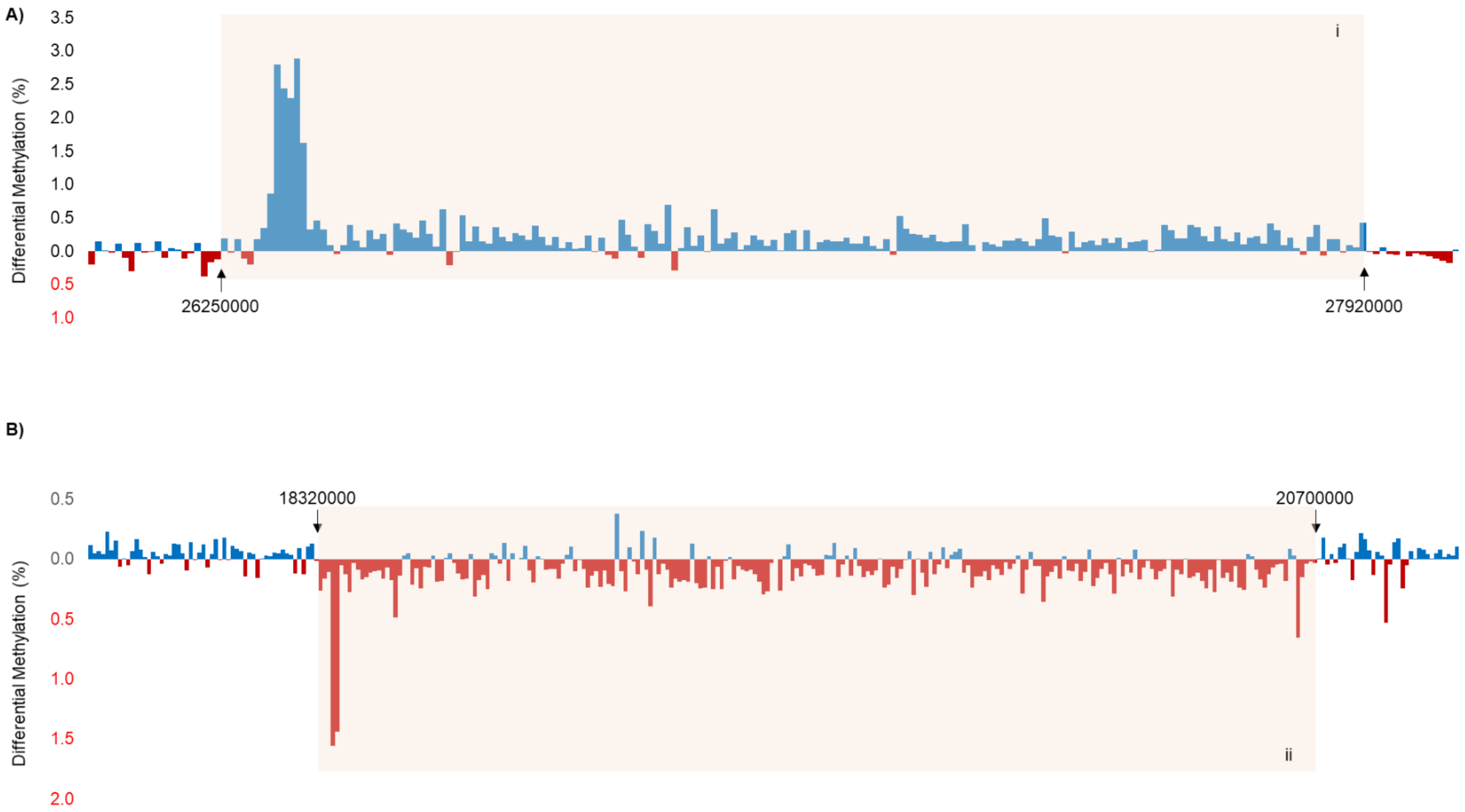
A)
3.5
3.0
2.5
2.0
1.5
1.0
0.5
0.0
0.5
1.0
Differential Methylation (%)
i
26250000
27920000
B)
0.5
0.0
0.5
1.0
1.5
2.0
Differential Methylation (%)
18320000
20700000
ii

Figure. S7

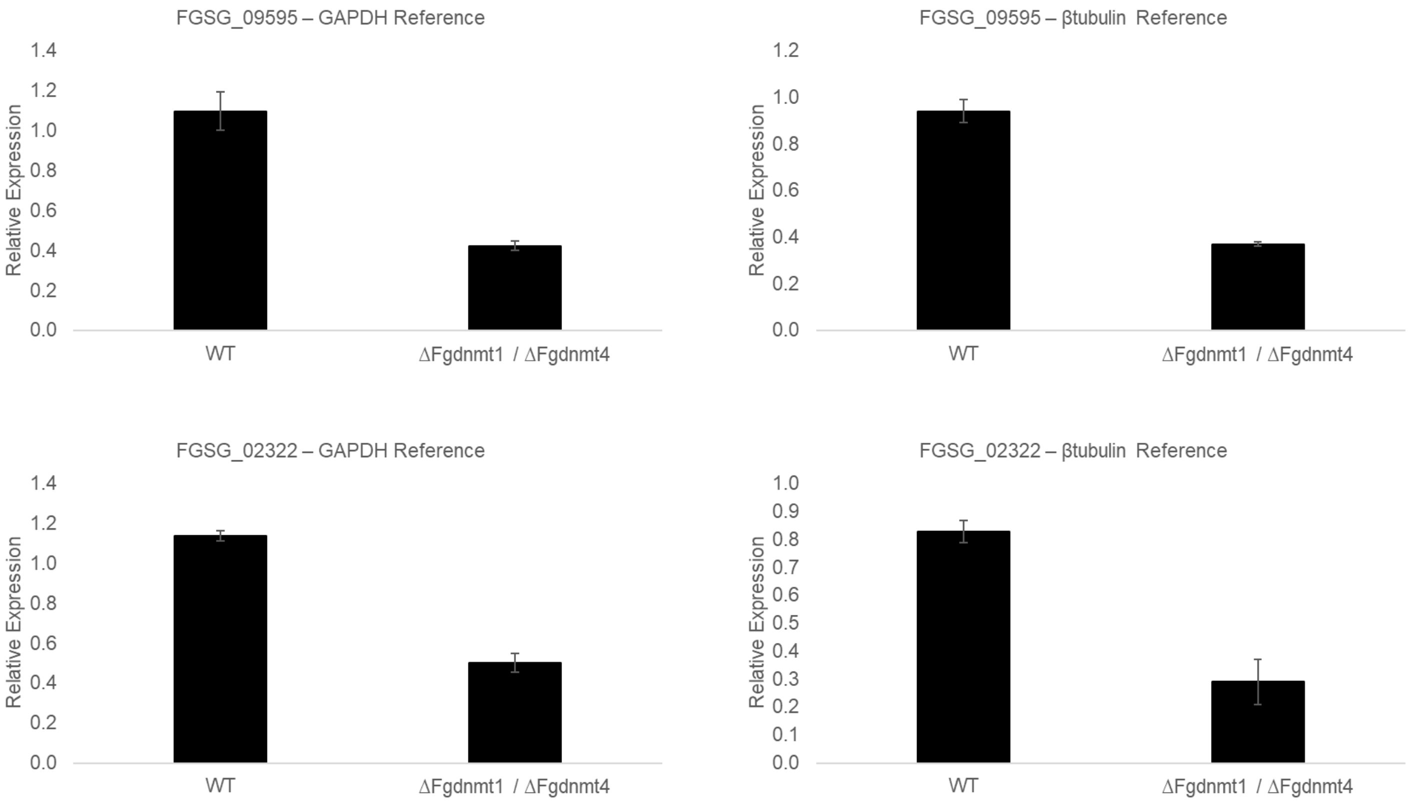
FGSG_09595 – GAPDH Reference
Relative Expression
1.4
1.2
1.0
0.8
0.6
0.4
0.2
0.0
WT
ΔFgdnmt1 / ΔFgdnmt4
FGSG_09595 – βtubulin Reference
Relative Expression
1.2
1.0
0.8
0.6
0.4
0.2
0.0
WT
ΔFgdnmt1 / ΔFgdnmt4
FGSG_02322 – GAPDH Reference
Relative Expression
1.4
1.2
1.0
0.8
0.6
0.4
0.2
0.0
WT
ΔFgdnmt1 / ΔFgdnmt4
FGSG_02322 – βtubulin Reference
Relative Expression
1.0
0.9
0.8
0.7
0.6
0.5
0.4
0.3
0.2
0.1
0.0
WT
ΔFgdnmt1 / ΔFgdnmt4

Figure. S8

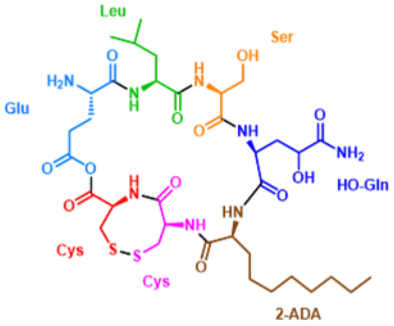

Gramillin A

Chemical Formula: $C_{35}H_{58}N_8O_{12}S_2$
Exact Mass: 846.3616

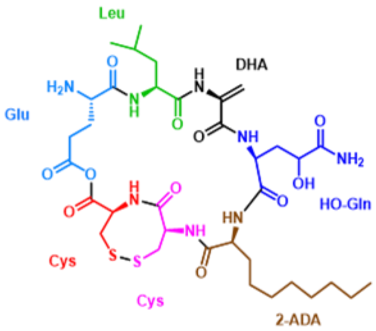

Gramillin E

Chemical Formula: $C_{35}H_{56}N_8O_{11}S_2$
Exact Mass: 828.3510

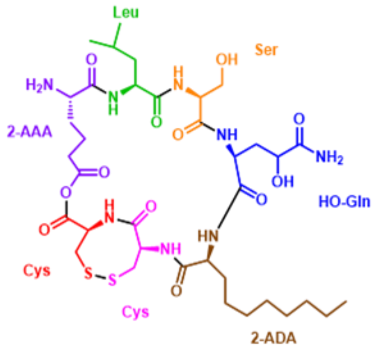

Gramillin B

Chemical Formula: $C_{36}H_{60}N_8O_{12}S_2$
Exact Mass: 860.3772

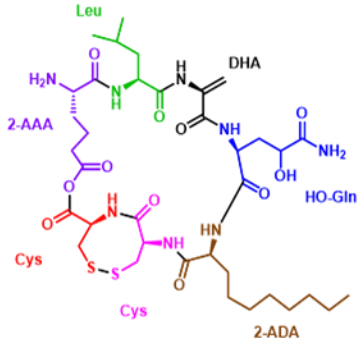

Gramillin F

Chemical Formula: $C_{36}H_{58}N_8O_{11}S_2$
Exact Mass: 842.3666
