# Supplementary material for: DNA Methylation Is Responsive to the Environment and Regulates the Expression of Biosynthetic Gene Clusters, Metabolite Production, and Virulence in Fusarium graminearum
Source: Front Fungal Biol. 2021 Jan 15;1:614633. doi: 10.3389/ffunb.2020.614633 (PMC10512235; doi:10.3389/ffunb.2020.614633)
Supplement: Supplementary Table 1 — DNMTs from F. graminearum share identity with RID2 and DIM-2 from Neurospora tetrasperma. Protein sequences were obtained from Uniprot (https://www.uniprot.org/) under accession numbers Q8NJV8 (NtRID), G4UYB9 (NtDIM-2), I1RWH8 (FGSG_08648) and I1S1Y7 (FGSG_10766). The sequences were subjected to reciprocal protein-protein BLASTp searches on NCBI (https://blast.ncbi.nlm.nih.gov/Blast.cgi). [file Data_Sheet_6.pdf]

Table S1. DNMTs from *F. graminearum* share identity with RID2 and DIM-2 from *Neurospora tetrasperma*. Protein sequences were obtained from Uniprot (https://www.uniprot.org/) under accession numbers Q8NJV8 (*NtRID*), G4UYB9 (*NtDIM-2*), I1RWH8 (FGSG_08648) and I1S1Y7 (FGSG_10766). The sequences were subjected to reciprocal protein-protein BLASTp searches on NCBI (https://blast.ncbi.nlm.nih.gov/Blast.cgi).

***F. graminearum* Strain**	*Nt* RID (Q8NJV8) Identity (e-value)	*Nt* DIM-2 (G4UYB9) Identity (e-value)
FGSG_08648 (I1RWH8)	35.7% (2e-95)	23.6% (2e-7)
FGSG_10766 (I1S1Y7)	26% (8e-13)	48.3% (0.0)

Table S2. Whole genome bisulfite sequencing statistics for WT and ∆*FgDim-2*/∆*FgRid* strains sequenced using Illumina HiSeq X PE150. Sequencing reads were deposited under accession # PRJNA587083 and mapped to the reference genome (Accession # SPRZ00000000) using CLC-Genomics Workbench.

		WT					∆*FgDim-2*/∆*FgRid*					
		PN		NPN			PN			NPN		
Sample	Sequencing Statistics	BR 1	BR 2	BR 1	BR 2	BR 3	BR 1	BR 2	BR 3	BR 1	BR 2	BR 3
Genome size	36,766,638											
%C+G	47.91											
Gene numbers	14190											
Method	PE[b]											
Conversion rate(%)[a]	99.5											
Total reads (Mbp)		68,974,006	65,251,418	66,751,428	62,125,272	59,077,414	60,359,444	66,775,820	47,251,660	70,047,640	70,366,392	70,048,580
Number of mapped reads (Mbp)		61,192,341	58,245,915	58,451,778	55,195,708	52,689,907	54,702,044	59,086,782	41,952,959	62,363,043	62,072,370	62,541,206
Read Length (bp)[c]		128.76	129.2	128.85	127.77	127.37	128.84	128.83	128.77	128.75	128.74	128.68
Mapping efficiency (%)		88.72	89.26	87.57	88.85	89.19	90.8	88.63	88.93	89.22	88.37	89.51
Coverage (%)		100	100	100	100	100	100	100	100	100	100	100
Mean sequencing depth per strand		212.05	202.87	201.42	187.21	177.66	189.85	203.7	144.65	213.41	212.74	214.51

BR: Biological replicates; PN: Preferred Nutrient conditions, and NPN: Non-Preferred nutrient conditions

[a] Conversion Rate: the conversion rate of BS-seq for F. graminearum is calculated from an un-methylated lambda DNA added to the BS-seq library

[b] PE: Paired End Reads

[c] Post Trimming

Table S3: DNA methylation is present under all three cytosine contexts, CpG, CHG and CHH in WT and ∆*FgDim-2*/∆*FgRid* strains, under both 24 hrs PN and 6 hrs NPN environmental conditions. Methylation level remained consistent between strains and environmental conditions. Methylation level is defined as the proportion of methylation at any given site in a population.

	WT					∆*FgDim-2*/∆*FgRid*					
	PN		NPN			PN			NPN		
Cytosine Context	BR 1	BR 2	BR 1	BR 2	BR 3	BR 1	BR 2	BR 3	BR 1	BR 2	BR 3
CpG(%)	4.45	4.72	4.97	5.11	5.29	4.88	4.64	6.00	4.62	4.77	4.50
CHG(%)	4.45	4.71	4.94	5.14	5.38	4.89	4.65	6.00	4.63	7.72	4.53
CHH(%)	4.68	4.90	5.10	5.52	5.80	5.20	4.73	6.03	4.95	4.97	4.86

BR: Biological replicates; PN: Preferred nutrient conditions; NPN: non-preferred nutrient conditions.

Table S4. DNA methylation exists in WT and Δ*FgDim-2*/Δ*FgRid* strains with minor differences between strains at the genome wide level. DNA methylation density was defined as the number of methylated cytosine as a percentage of the total cytosine. DNA methylation was predominantly identified in the asymmetrical CHH context in both strains.

	WT					Δ*FgDim-2*/Δ*FgRid*					
	PN		NPN			PN			NPN		
Cytosine Context	BR 1	BR 2	BR 1	BR 2	BR 3	BR 1	BR 2	BR 3	BR 1	BR 2	BR 3
CpG	0.41	0.44	0.6	0.35	0.36	0.72	0.37	0.42	0.29	0.43	0.4
CHG	0.37	0.39	0.52	0.32	0.32	0.6	0.33	0.36	0.27	0.38	0.37
CHH	1.3	1.39	1.71	1.07	1.09	1.94	1.16	1.21	0.95	1.33	1.26
Average	2.15		2.11			2.37			1.89		

BR: Biological replicates; PN: Preferred Nutrient conditions, and NPN: Non-Preferred nutrient conditions. Values represent methylation density (#5mC/#GenomicC*100).

Table S5. Primers Used in This Study

Primer Name	Sequence (5'-3')	
P1 - DIM2	gggtttaaugcagcctatcctcatgaagtga	KO
P2 - DIM2	ggacttaaugtcctatgttaatgaatatgcacc	KO
P3 - DIM2	ggcattaaugtcggaaacagtgtcgcgc	KO
P4 - DIM2	ggtcttaauaccccaacatcgtttc	KO
P1 - RID	gggtttaaugtggattagtgtgttgaggaac	KO
P2 - RID	ggacttaauccaaggtaggtagcaacgaatg	KO
P3 - RID	ggcattaautgattgaggcgcggagtatc	KO
P4 - RID	ggtcttaauaggaaatgaaggagcccgtg	KO
P5 - DIM2	ggacttaaucgagaacattcttggtttgg	Compl
P6 - DIM2	gggtttaauctactcatttgaaacgctg	Compl
P5 - RID	ggacttaaugtgaacatggatttctgatc	Compl
P6 - RID	gggtttaauttaggtcaattcgatc	Compl
DIM2 Int. F	gtggtcgatctgagccttgc	Confirm
DIM2 Int. R	gttccgccattggatcac	Confirm
RID Int. F	caagacgaagcaaagctca	Confirm
RID Int. R	atgtacggatgcatgagtgt	Confirm
HYG F	agctgcgccgatggtttctacaa	Confirm
HYG R	gcgcgtctgctgctccatacaa	Confirm
Gen F	tcatcaatcccagccttttc	Confirm
Gen R	cagtcgatgaatccagaaaagc	Confirm
TRI6 ORF F	atgatttacatggaggccg	Confirm
TRI6 ORF R	acacttatgtatccgcctatagtg	Confirm
gpdA F	gaagtggaaaggctggtgtg	Confirm
gpdA R	ataagggatgggaaggatgg	Confirm
FGSG_09530 F (*β tubulin*)	gttgatctccaagatccgtg	qPCR
FGSG_09530 R (*β tubulin*)	catgcaaatgtcgtagaggg	qPCR
FGSG_16627 F (*GAPDH*)	tgacttgactgttcgcctcgagaa	qPCR
FGSG_16627 R (*GAPDH*)	atggaggagttggtgttgccgtta	qPCR
DIM2 qPCR F	ggaggtggtatcgcgatgcg	qPCR
DIM2 qPCR R	tcgatcgagcccaagaatgg	qPCR
RID qPCR F	actcgtcttctctcaccgga	qPCR
RID qPCR R	aaacactgtggctcaaacgc	qPCR
FGSG_02322 qPCR F	aagtgatgtgcctacgggtg	qPCR
FGSG_02322 qPCR R	tcgcaacatcaatcccgtca	qPCR
FGSG_09595 qPCR F	ttacaccattcccctcgtgc	qPCR
FGSG_09595 qPCR R	gtgtcgggtttgagggtgat	qPCR
**Primers used in BSseq Analysis**		
Loci_Contig1_9033981-9034488 R	gtatttataaatagatttaaagttataaagt	BSseqAnalysis
Loci_Contig1_9033981-9034488 F	aacatactacactttcctaa	BSseqAnalysis
Loci_Contig6_127178-127396 F	gttaatggttttgggaatggtat	BSseqAnalysis
Loci_Contig6_127178-127396 R	ataaaaacctattaaaaaataataaaaatt	BSseqAnalysis
M13F	tgtaaaacgacggccagt	BSseqAnalysis
M13R	caggaaacagctatgacc	BSseqAnalysis
